# Comparative Evaluation of hiPSC-Derived Brain Organoids as Platforms for Assessing Thyroid Hormone System Disrupting Chemicals

**DOI:** 10.3390/cells15110963

**Published:** 2026-05-22

**Authors:** Valeria Fernandez Vallone, Lina Hellwig, Eddy Rijntjes, Nicolai von Kügelgen, Rajas Sane, Robert Opitz, Peter Kühnen, Josef Köhrle, Philipp Mergenthaler, Harald Stachelscheid

**Affiliations:** 1 Core Unit Pluripotent Stem Cells and Organoids, Berlin Institute of Health, Charité–Universitätsmedizin Berlin, Charitéplatz 1, 10117 Berlin, Germany; harald.stachelscheid@bih-charite.de; 2 Center for Stroke Research Berlin, Charité–Universitätsmedizin Berlin, Charitéplatz 1, 10117 Berlin, Germany; lina.hellwig@charite.de (L.H.);; 3 Department of Neurology with Experimental Neurology, Charité–Universitätsmedizin Berlin, Charitéplatz 1, 10117 Berlin, Germany; 4 Institut für Experimentelle Endokrinologie, Charité–Universitätsmedizin Berlin, Charitéplatz 1, 10117 Berlin, Germany; eddy.rijntjes@charite.de (E.R.); nicolai.von-kuegelgen@bih-charite.de (N.v.K.); rajas.sane@charite.de (R.S.); josef.koehrle@charite.de (J.K.); 5 Core Unit Bioinformatics, Berlin Institute of Health, Charité–Universitätsmedizin Berlin, Charitéplatz 1, 10117 Berlin, Germany; 6 Department for Paediatric Endocrinology and Diabetology, Charité–Universitätsmedizin Berlin, Charitéplatz 1, 10117 Berlin, Germany; robert.opitz@charite.de (R.O.); peter.kuehnen@charite.de (P.K.); 7 German Center for Child and Adolescent Health (DZKJ), Partner Site Berlin, Augustenburger Platz 1, 13353 Berlin, Germany; 8 Radcliffe Department of Medicine, University of Oxford, Oxford OX3 9DS, UK

**Keywords:** brain organoids, thyroid hormone system disruption, NAMs, validation, endpoints

## Abstract

**Highlights:**

**What are the main findings?**

- T3-responsive gene expression, T3 metabolism, and SOX2-positive progenitor populations are informative endpoints for detecting THSDC effects in hiPSC-derived brain organoids, as shown by altered T3-dependent responses to silychristin and iopanoic acid, particularly under chronic exposure.
- Differentiation-stage quality control improves the interpretability, reproducibility, and assay readiness of distinct hiPSC-derived brain organoid platforms.

**What are the implications of the main findings?**

- hiPSC-derived brain organoids have potential as human-relevant NAMs to study thyroid hormone system disruption during early brain development.
- Standardized workflows with defined QC checkpoints are essential for advancing brain organoid models toward reproducible toxicology testing and future regulatory applications.

**Abstract:**

Thyroid hormones (THs) are essential regulators of human brain development, and disrupted TH availability during pregnancy or early life is linked to adverse neurodevelopmental outcomes. Concerns that environmental chemicals interfere with TH signalling have increased the need for human-relevant in vitro systems to identify thyroid hormone system-disrupting chemicals (THSDCs) for risk assessment. Here, we compared two human-induced pluripotent stem cell (hiPSC)-derived brain organoid models for THSDC assessment: (i) human cortical organoids (COs) generated by unguided differentiation, offering higher architectural complexity but lower throughput; and (ii) neural stem cell-derived organoids (NSCOs), designed for scalability with reduced cellular diversity. Both models expressed key TH handling components, including the transporter *SLC16A2* (MCT8) and the inactivating enzyme *DIO3*. Using LC–MS/MS, we show that exogenous T3 is depleted from culture media and metabolized to 3,3′-T2 and 3′-T1 in both models, alongside upregulation of T3-responsive genes (*HR*, *KLF9*, *DIO3*, *SEMA3C*). Pulse and chronic co-exposures to reference disruptors iopanoic acid (IA, deiodinase inhibitor) and silychristin (SC, MCT8 inhibitor) altered T3 metabolism and modulated T3-responsive transcriptional endpoints. In NSCOs, high-content imaging revealed treatment-associated changes in cell composition, with chronic T3 reducing the SOX2-positive progenitor pool and THSDCs blocking this effect. Together, these findings provide a framework for organoid qualification—linking TH handling, transcriptomic responsiveness, and scalable phenotypic readouts—as a necessary step toward model validation and implementation of brain organoids in THSDC risk assessment pipelines.

## 1. Introduction

Thyroid hormones (THs), principally thyroxine (T4) and 3,3′-5-triiodothyronine (T3), are indispensable regulators of human neurodevelopment. Inadequate TH action during brain development can result in largely irreversible neurocognitive impairment, underscoring that appropriate TH supply is a prerequisite for normal brain maturation [1,2].

TH signalling influences proliferation and fate decisions of neural progenitors, neuronal migration and layer formation, neurite outgrowth, synaptogenesis, gliogenesis, and myelination [2,3]. Importantly, TH action in the developing brain is not determined solely by circulating hormone concentrations. Instead, local control mechanisms shape when and where neural cells experience TH signalling. These mechanisms include transmembrane transporters (e.g., MCT8), intracellular deiodinases that activate (DIO2) or inactivate (DIO3) iodothyronines, and cell-type-specific receptor expression [4,5,6].

A wide array of environmental, nutritional, and industrial chemicals can interfere with the TH system. Many are endocrine-disrupting chemicals (EDCs), broadly defined as exogenous agents that alter endocrine function by affecting hormone synthesis, release, transport, metabolism, binding, activity, or elimination [6]. TH system-disrupting chemicals (THSDCs) include certain plasticizers, flame retardants, alkylphenols, dioxins and related persistent organic pollutants, some metals, and per- and polyfluoroalkyl substances (PFAS) and are present in the environment as mixtures [6,7]. Prenatal exposure is of special concern because maternal–fetal transfer can occur for many chemicals during sensitive developmental windows [6].

Many THSDCs alter serum TH concentrations and/or affect TH signalling in vitro and in vivo, yet the relationship between systemic changes and tissue-level TH action—particularly in the fetal brain—remains insufficiently characterized for hazard identification [1,8,9]. This gap matters because systemic endpoints (e.g., serum T4/TSH) may not reflect local effects governed by transport, deiodination, and receptor occupancy [2,5,10,11,12,13]. Conversely, tissue-specific disruption could occur without marked systemic changes in hormone concentrations. Therefore, better metrics of TH action within developing human neural tissue are needed to evaluate how exposures affect the developing brain more directly. Current regulatory testing strategies only partially address this need. Within the OECD endocrine disruptor testing and assessment framework, TH system disruption still relies on endpoints such as weight, TH serum concentrations and thyroid histopathology in rodents [14,15,16,17,18].

This scenario coincides with a broader shift in toxicology toward new approach methodologies (NAMs). NAMs encompass in silico, in chemico, in vitro, and ex vivo methods and their implementation strategies, aiming to reduce reliance on animal testing while providing mechanistic information for chemical safety assessment [19,20]. Regulatory bodies have emphasized that NAMs used for safety decision-making must demonstrate validity and reliability for their intended context of use and should be at least as informative as existing guideline studies (FDA Science Board’s NAMs 2024). NAMs that capture TH-mediated adverse effects in peripheral target tissues—especially developmental neurotoxicity—remain scarce, and they are far from being validated assays [17,21,22,23].

Human-relevant in vitro systems that model neurodevelopment offer a route to close this gap. Ex vivo tissue explants preserve aspects of native architecture but are constrained by limited availability, restricted developmental windows, and donor-to-donor variability. Additionally, species differences can complicate extrapolation from rodents to humans. These limitations have motivated the development of human-induced pluripotent stem cell (hiPSC)-derived neural models for THSDC identification and assessment [18,24].

Organoid protocols generate 3D tissues that self-organize into neuroepithelia and produce multiple neuronal populations, capturing aspects of human in vivo cytoarchitecture and developmental gene expression trajectories [25,26]. Neural organoids have also been increasingly applied to toxicology as advanced platforms to investigate mechanisms of neurotoxicity and to bridge between reductionist cell assays and whole-animal studies [7,27,28].

At the same time, translating brain organoids into fit-for-purpose toxicological assays requires confronting well-recognized challenges. Organoids can exhibit substantial heterogeneity and batch-to-batch variability driven by stochastic self-organization and differences in starting cell populations or culture conditions. Methods can be labour-intensive, time-consuming, and difficult to scale [29]. Biological limitations—including incomplete maturation, lack of vasculature, diffusion constraints and variability in regional patterning—intersect directly with assay development requirements. For regulatory or screening contexts, organoid systems must be standardized, quality-controlled (critical checkpoints), and benchmarked with reference compounds.

Multiple strategies can improve organoid readiness for THSDC testing. Protocol choices can deliberately trade biological complexity for robustness. Systematic quality control (QC)—combining morphology, viability, marker expression, and omics-based benchmarks—can identify non-compliant cultures early and facilitate cross-laboratory transferability [30]. Recent work proposes practical QC frameworks tailored to brain cortical organoid workflows [31,32]. Finally, selecting endpoints mechanistically anchored to TH biology (e.g., TH-responsive transcriptional programmes, neurogenesis and gliogenesis pathways, metabolic readouts and differentiation state) can enhance interpretability and facilitate integration with existing TH system-related mechanistic assays.

In this study, we aimed to develop hiPSC-derived brain organoid-based assays to assess TH system disruption. For this, we used two organoid platforms with distinct trade-offs: (i) cerebral organoids (CO) that develop complex cytoarchitecture and cellular diversity but are labour-intensive and prone to heterogeneity, and (ii) neural stem cell-derived organoids (NSCO) that are less complex but more scalable and potentially more reproducible. We first defined a set of endpoints—including transcriptome-wide responses, TH depletion and metabolization, and cell-type composition—as sensitive readouts of THSDC effects. We then used those endpoints to evaluate the reference THSDC representing different modes of action.

By integrating standard operating procedures (SOPs) and QC checkpoints for both models during differentiation, we propose a framework for reproducibility and standardization. To our knowledge, this is the first study that defines a set of endpoints to assess THSDC in brain development using reference compounds and different exposure schedules on brain organoids derived from multiple donors. Our work seeks to provide direct measures of altered TH action in developing human neural tissue, clarify how model complexity and exposure design influence detectability of TH-mediated effects, and contribute practical guidance for establishing organoid-based NAMs that are robust enough to enter pre-validation pipelines and complement ongoing OECD efforts to build mechanistic thyroid test batteries.

## 2. Materials and Methods

### 2.1. hiPSC Lines and Culture

hiPSC lines, derived from different donors, BIHi001-B (male; hPSCreg: https://hpscreg.eu/cell-line/BIHi001-B, accessed on 15 January 2026), BIHi005-A (male; https://hpscreg.eu/cell-line/BIHi005-A (accessed on 15 January 2026)), and BIHi250-A (female; https://hpscreg.eu/cell-line/BIHi250-A (accessed on 15 January 2026)) were generated in-house by the Berlin Institute of Health Core Unit Pluripotent Stem Cells and Organoids (BIH-CUSCO). The hiPSC line UCSFi001-A-37 (SOX2-GFP-reporter, male, https://hpscreg.eu/cell-line/UCSFi001-A-37 (accessed on 15 January 2026)) was acquired from the Allen Institute for Cell Science and the master bank used, produced and provided by BIH-CUSCO. The hiPSC line HMGUi001-A (female; https://hpscreg.eu/cell-line/HMGUi001-A (accessed on 15 January 2026)) was kindly provided by Heiko Lickert from Helmholtz Zentrum München, Germany [33].

Prior to differentiation, hiPSCs were maintained in Essential 8 (E8) medium on Geltrex (ThermoFisher Scientific, A1413202, Waltham, MA, USA)-coated plates and passaged chemically using 0.5 mM EDTA at ~70% confluency. All maintenance culture details were followed according to good practices as previously described [34]. This study was conducted under ethical approvals EA2/013/16 and EA2/145/22 from the Charité Ethics Commission.

### 2.2. Triiodothyronine (T3), Silychristin (SC) and Iopanoic Acid (IA) Stock Solution Preparation

3,3′,5-Triiodo-L-thyronine sodium salt (T3) (Sigma-Aldrich, T6397, St.Louis, MO, USA) was dissolved in 100% ethanol (Carl Roth, 5054.3, Karlsruhe, Germany) to prepare a 3 mM stock solution (2 mg/mL), as recommended by the manufacturer. Stock aliquots were stored at −80 °C and subjected to a maximum of three freeze–thaw cycles. For use, an intermediate 1:1000 working stock dilution (3 µM) was prepared freshly in the corresponding culture medium. This intermediate solution was then used to prepare culture media at the indicated final T3 concentrations.

Silychristin [35] (SC; phyproof^®^ reference substance; CAS 33889-69-9, PhytoLab, Vestenbergsreuth, Germany, purchased fromSigma-Aldrich, PHL89281, St.Louis, MO, USA) was dissolved in DMSO (Carl Roth, A994.1, Karlsruhe, Germany) to generate a 10 mM stock solution (10 mg in 2.073 mL). Aliquots were stored at −80 °C and subjected to a maximum of three freeze–thaw cycles. SC was added freshly to the culture media at each medium preparation.

Iopanoic acid [36] (IA; CAS 96-83-3) (Selleckchem, S5497; Batch No. 1, Houston, TX, USA) was dissolved in DMSO (Carl Roth, A994.1, Karlsruhe, Germany) to generate a 30 mM stock solution (25 mg in 1.378 mL). Aliquots were stored at −80 °C and subjected to a maximum of three freeze–thaw cycles. IA was added freshly to culture media at each medium preparation.

SC and IA have higher solubility in DMSO than ethanol, allowing lower solvent proportion carry-over in treatments. The same lot and stock solutions of all reagents were used throughout the entire study.

### 2.3. Cerebral Organoid (CO) Generation

COs were generated using the STEMdiff^TM^ Cerebral Organoid Kit (STEMCELL Technologies, 08570, Vancouver, BC, Canada) according to the manufacturer’s instructions unless stated otherwise. More details on the differentiation procedure can be found in the Supplementary Information, extended methods.

### 2.4. CO Treatment and Experimental Design

Early and late exposure windows were selected based on CO differentiation dynamics, to compare TH/THSDC effects during the progenitor-rich phase (early) with the more differentiated stage (late) after initial layering. These exposure windows were not intended to model specific clinical periods directly, but rather to adapt to technical constraints. Both windows were similar in length. The early window started right after CO extracellular matrix capsule removal (day 23).

**Early exposure:** Until day 23, COs from the early exposure group were cultured in maturation medium (STEMdiff^TM^ Cerebral Organoid Kit, STEMCELL Technologies, 08570, Vancouver, BC, Canada). On day 23 of differentiation, COs were transferred to 6-well plates (8 COs per well) in 4 mL per well of cerebral organoid differentiation medium (COD) composed of 50% Advanced DMEM/F12 (ThermoFisher Scientific, 12634-010, Waltham, MA, USA) and 50% neurobasal medium (ThermoFisher Scientific, 21103-049, Waltham, MA, USA), supplemented with 1X B27 supplement (ThermoFisher Scientific, 17504-044, Waltham, MA, USA), 0.5X N2 supplement (ThermoFisher Scientific, 17502-048), 1X Glutamax (ThermoFisher Scientific, 35050038), 1X NEA (ThermoFisher Scientific, 11140050, Waltham, MA, USA), 2.5 µg/mL Insulin (CS Bio, CS9212, Menlo Park, CA, USA), 45 µM 2-Mercapto-ethanol (ThermoFisher Scientific, 31350010, Waltham, MA, USA) and 1X antibiotic/antimycotic (ThermoFisher Scientific, 15240062, Waltham, MA, USA). For chronic exposure, COs were pre-treated with the reference THSDC for 24 h (day 23–24), followed by co-incubation with T3 in the continued presence of the THSDC from day 24 to day 40. For pulse exposure, COs were pre-treated with the reference THSDC for 24 h on day 37, followed by co-incubation with T3 in the continued presence of the THSDC from day 38 to day 40 (48 h). For all conditions, day 38 was the last full medium change. Appropriate vehicle controls (DMSO) were included. On day 40, culture supernatant was collected and split into two technical replicate aliquots (1.5 mL each) and stored at −20 °C for subsequent LC–MS/MS analysis. In parallel, three COs per condition were collected individually, washed in PBS, and lysed in lysis buffer (Aurum^TM^ Total RNA Mini Kit; Bio-Rad, 732-6820, Hercules, CA, USA) supplemented with 1% 2-mercaptoethanol (Sigma-Aldrich, M6250) and stored at −80 °C until use. Remaining COs were washed in PBS and fixed overnight in 4% PFA (Carl Roth, 0335.2, Karlsruhe, Germany). The following day, fixed COs were washed and stored in PBS at 4 °C until further use. Refer to Figure 2A for visualization of the treatment scheme.

**Late exposure:** Until day 40, COs from the late exposure group were cultured in maturation medium (STEMdiff^TM^ Cerebral Organoid Kit, STEMCELL Technologies, 08570, Vancouver, BC, Canada). On day 40, COs were transferred to 6-well plates (6 COs per well) in 4 mL of COD per well. For the chronic exposure, COs were pre-treated with the reference THSDC for 24 h (day 40–41), followed by co-incubation with T3 in the continued presence of the THSDC from day 41 to day 60. For pulse exposure, COs were pre-treated with the reference THSDC for 24 h on day 57, followed by co-incubation with T3 in the continued presence of the THSDC from day 58 to day 60 (48 h). For all conditions, day 58 was the last full medium change. Appropriate vehicle controls (DMSO) were included. On day 60, culture supernatant was collected and split into two technical replicate aliquots (1.5 mL each) and stored at −20 °C for subsequent LC–MS/MS analysis. Three COs per condition were collected individually, washed in PBS, and lysed in lysis buffer (Aurum^TM^ Total RNA Mini Kit; Bio-Rad, 732-6820, Hercules, CA, USA) supplemented with 1% 2-mercaptoethanol (Sigma-Aldrich, M6250, St. Louis, MO, USA) and stored at −80 °C until use. Remaining COs were washed in PBS and fixed overnight in 4% PFA (Carl Roth, 0335.2, Karlsruhe, Germany). The following day, fixed COs were washed and stored in PBS at 4 °C until further use.

All medium changes were complete (100%) with fresh supplementation of T3 and reference compounds at each exchange. All incubations were performed at 37 °C, 5% CO_2_, on an orbital shaker at 60 rpm. 

**Control condition:** Unless stated otherwise, this was always COs cultured in COD without the addition of T3 or reference THSDC.

### 2.5. Neural Stem Cells (NSC) Differentiation and Banking

NSCs were differentiated in 6-well plates and banked as previously described [37]. For banking, NSCs were aliquoted at 2,000,000 cells per cryovial. Out of 3 plates of 6-well plates, we froze 50–60 cryovials. We generated 1 bank for NSCs derived from BIHi250-A hiPSC line and 1 bank for NSCs derived from HMGUi001-A hiPSC line.

### 2.6. NSC Quality Control

Prior to banking at day 7, NSC marker expression was assessed by FACS, as previously described [37]. Co-expression of SOX2/PAX6 and SOX1/NESTIN above 90% was considered the acceptance criterion for bank quality.

Post banking production, NSC quality was assessed in a thawing test, as previously described [37]. Viability > 70% was considered acceptable. Thawed NSCs were cultured and subjected to an additional QC by immunofluorescence. Details can be found in the Supplementary Information, extended methods. Expression of SOX2/PAX6/SOX1 > 90% was considered acceptable.

### 2.7. Neural Stem Cell-Derived Organoids (NSCO) Generation

NSCOs were generated from NSCs obtained from quality-controlled cryobanks, as previously described.

**Figure 1:**
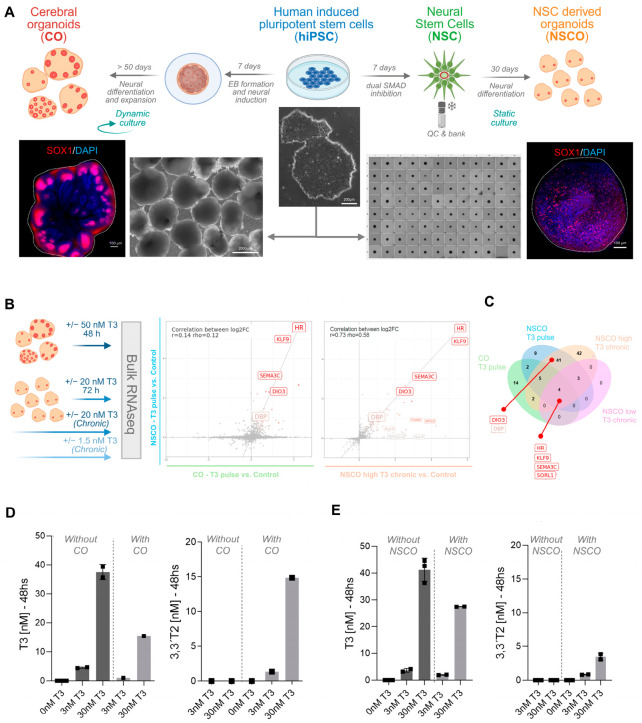
hiPSC-derived cerebral organoids (COs) and neural stem cell-derived organoids (NSCOs) are responsive to triiodothyronine (T3) in vitro. (**A**) Schematic of differentiation workflows generating COs (left; dynamic culture) and NSCOs (right; static culture via an intermediate neural stem cell [NSC] stage). Representative bright-field images show hiPSCs prior to differentiation, COs at day 50, and NSCOs at day 30. Immunofluorescence images of vibratome sections illustrate organoid cytoarchitecture, with SOX1 marking neural progenitors and DAPI staining nuclei. Scale bars: hiPSC, 200 µm; CO bright-field, 2000 µm; immunofluorescence, 100 µm. (**B**) Bulk RNA-sequencing experimental design and transcriptomic response to T3. Left, schematic of exposure conditions prior to RNA collection (CO: ±50 nM T3 for 48 h [pulse], *n* = 1 biological replicate -BIHi005-A- and *n* = 3 pools of 3 different CO technical replicates; NSCO: ±20 nM T3 for 72 h [pulse] or chronic exposure at 20 nM [high-T3] or 1.5 nM [low-T3], *n* = 1 biological replicate -BIHi250-A- and *n* = 3 pools of 6 different NSCO technical replicates). Middle, concordance plots comparing log2 fold-changes of genes in CO T3 pulse vs. control and NSCO T3 pulse vs. control (left plot) and comparing NSCO high-T3 chronic vs. control with NSCO T3 pulse vs. control (right plot). Selected shared upregulated genes are annotated. (**C**) Venn diagram showing overlap of upregulated genes (vs. control) across conditions (CO T3 pulse, NSCO T3 pulse, NSCO high-T3 chronic, and NSCO low-T3 chronic). Selected upregulated shared genes are highlighted. (**D**,**E**) LC–MS/MS quantification of T3 and its metabolite 3,3′-diiodothyronine (3,3′-T2) in culture supernatants 48 h after T3 pulse exposure in CO (HMGUi001-A, day 60; 6-well format) (**D**) and NSCO (BIHi250-A, day 50; 6-well format) (**E**). Bars represent mean ± SD; points indicate individual wells from the same experiment. *Schemes were created using BioRender.*

NSCOs generated in *dynamic culture* were used for exploratory experiments for assessment of T3 consumption and metabolism (Figure 1 and Figure S10B) [38]. Briefly, NSCOs were transferred on day 15 to 6-well plates and cultured on an orbital shaker (65 rpm) in neural differentiation medium (NDM) consisting of neurobasal medium (ThermoFisher Scientific, 21103-049, Waltham, MA, USA) supplemented with 20 ng/mL NT-3 (PeproTech, 450-03, Rocky Hill, NJ, USA), 20 ng/mL BDNF (PeproTech, 450-02, Rocky Hill, NJ, USA), 1X B27 (ThermoFisher Scientific, 17504-044, Waltham, MA, USA), 1X GlutaMAX (ThermoFisher Scientific, 35050038, Waltham, MA, USA), and 1X antibiotic/antimycotic (ThermoFisher Scientific, 15240062, Waltham, MA, USA). NDM was supplemented additionally with 1% Geltrex. Under these conditions, NSCOs increased in size and were maintained until day 50, when supernatants were collected for downstream LC–MS/MS analysis. For these assays, 10 organoids per well were cultured in 4 mL NDM supplemented with the indicated concentrations of T3 and reference THSDCs (as specified in the corresponding figures).

NSCOs generated in *static culture* as previously described [38] were cultured in NDM and used as the main dataset for which treatments were conducted in 96-round ULA well-plates under static conditions.

All microscopy was performed using Leica DMi8 (Leica Microsystems, Wetzlar, Germany).

### 2.8. NSCO Treatment and Experimental Design

For chronic exposure, NSCOs were pre-treated with the reference THSDCs for 24 h starting on day 9 of differentiation (day 9–10), followed by co-incubation with T3 in the continued presence of the THSDC from day 10 to day 33. For pulse exposure, NSCOs were pre-treated with the reference THSDC for 24 h on day 29, followed by co-incubation with T3 in the continued presence of the THSDC from day 30 to day 33 (72 h). For all conditions, day 30 was the last full medium change. Appropriate vehicle controls (DMSO) were included.

On day 33, culture supernatants were collected into a “mirror” 96-well plate (ThermoFisher Scientific, 11510294, Waltham, MA, USA), maintaining the original well positions/coordinates of the NSCO culture plate to preserve sample identity and enable direct traceability during downstream ELISA analysis. Mirror plates were stored at −20 °C until ELISA was performed. For RNA extraction, three independent pools (6 NSCOs per pool) were collected, washed in PBS, and lysed in lysis buffer (Aurum^TM^ Total RNA Mini Kit; Bio-Rad, 732-6820, Hercules, CA, USA) supplemented with 1% 2-mercaptoethanol (Sigma-Aldrich, M6250, St. Louis, MO, USA) and stored at −80 °C until use. Remaining NSCOs were washed in PBS and fixed overnight in 4% PFA (Carl Roth, 0335.2, Karlsruhe, Germany). The following day, fixed NSCOs were washed and stored in PBS at 4 °C until further use.

All medium changes were complete (100%) with fresh supplementation of T3 and reference compounds at each exchange. All incubations were performed at 37 °C and 5% CO_2_ under static culture conditions.

**Control condition:** Unless stated otherwise, this was always NSCOs cultured in NDM without the addition of T3 or reference THSDC.

### 2.9. High-Content Imaging and Analysis (HCA)

**Acquisition Pipeline:** Imaging was carried out on an Opera Phenix high-content imager (Revvity) operated with Harmony v5.2 software. Microscope slides were loaded into a µ-Slide Microscopy Rack (Ibidi, 80030, Gräfelfing, Germany) with the coverslips face down oriented. Organoid sections were imaged in their entirety using the Preciscan workflow, which employs an initial low-magnification pre-scan using a 10× air objective (NA = 0.3) for object detection and localization, followed by a high-magnification re-scan using a 20× air objective (NA = 0.8). A non-confocal pre-scan processed image served as the basis for object detection. Detected image regions were then separated into individual objects, and morphological properties, including area and roundness, were computed. Objects with an area smaller than 10,000 µm^2^ were removed. The high-magnification re-scan was carried out confocally. DAPI signal was acquired with the 375 nm laser (80% power, 40 ms exposure), cCas3 was detected using a 488 nm laser (80% power, 40 ms exposure) and SOX2 signal was captured using a 647 nm laser (100% power, 40 ms exposure). Z-stacks were acquired with 1 µm inter-plane spacing across 20 planes, covering a total depth of 19 µm. Although z-positions were guided by the pre-scan data, a default starting height of 3 µm was defined for the first imaging plane.

Image Analysis Pipeline: Images were analysed with the ImageArtist software (v1.3.29, Revvity, Waltham, MA, USA); a detailed description of the analysis pipeline is provided in Supplementary Table S4. The mean fraction of cells positive for cCas3 or SOX2 was plotted for each treatment group and organoid replicate.

### 2.10. NSCO Size Measurement

ULA 96-well plates containing NSCO cultures were imaged in a high-throughput manner using an Incucyte^®^ SX5 system (Sartorius AG, Göttingen, Germany). Scans were acquired in phase-contrast using the Single Spheroid mode at 4× magnification. Image analysis was performed using the “Spheroid” analysis module with a minimum area filter of 100,000 µm^2^. Quantitative measurements were exported as .csv files.

### 2.11. RNA Extraction and DNAse Treatment

Total RNA from organoid samples stored in lysis buffer was extracted according to the manufacturer’s instructions using the Aurum^TM^ Total RNA Mini Kit (Bio-Rad, 732-6820, Hercules, CA, USA). Residual genomic DNA was removed by DNase treatment of 1 µg RNA using the DNA-free^TM^ Kit (Invitrogen, AM1906, Carlsbad, CA, USA) following the manufacturer’s instructions. RNA concentration was determined using a NanoDrop 2000 spectrophotometer (ThermoFisher, Waltham, MA, USA). For samples processed for bulk RNA-sequencing, concentration of total RNA was measured by Qubit^®^ RNA BR Assay Kit (ThermoFisher, Waltham, MA, USA), and RNA integrity/quality (RIN > 9) was assessed using the High Sensitivity RNA ScreenTape system (Agilent Technologies, 5067-5576, Santa Clara, CA, USA) and Tape Station 4150 (Agilent Technologies, Santa Clara, CA, USA).

### 2.12. Bulk RNA-Sequencing and Analysis

**CO samples** subjected to bulk RNA-sequencing were derived from BIHi005-A hiPSC line at day 76 of culture, in control conditions or exposed to a 50 nM T3 pulse for 48 h. Technical replicates *n* = 3 consisted of individual pools of 3 COs each, all from the same differentiation experiment. For these samples, bulk 3′ mRNA Illumina sequencing libraries were prepared from 100 ng total RNA using the QuantSeq 3′ mRNA-Seq V2 Library Prep Kit FWD with UDI 12 nt (Lexogen, Vienna, Austria), according to the recommendations of the manufacturer. The resulting libraries were analysed by the D1000 ScreenTape Assay (Agilent Technologies, Santa Clara, CA, USA), and concentration was measured by the Qubit^®^ dsDNA HS Assay Kit (ThermoFisher, Waltham, MA, USA). After equimolar pooling, libraries were sequenced paired-end 100 cycles on an Illumina NovaSeq Xplus 10B flow cell (Illumina, Inc., San Diego, CA, USA), targeting ~30 million reads per sample.

**NSCO samples** subjected to bulk RNA-sequencing were derived from BIHi250-A hiPSC line at day 33 of culture, in control conditions or exposed to (i) chronic 1.5 nM T3, (ii) chronic 20 nM T3, (iii) pulse 20 nM T3 for 72 h. Technical replicates *n* = 3 consisted of individual pools of 6 NSCOs each, all from the same differentiation experiment. For these samples, bulk RNA-sequencing was performed by Brooks Life Sciences Genewiz^®^ using poly(A) selection for mRNA enrichment, targeting depth of ~30 million reads per sample. Sequencing libraries were prepared using the NEBNext^®^ Ultra^TM^ RNA Library Prep Kit for Illumina (New England Biolabs, E7770, Ipswich, MA, USA). Sequencing libraries were validated using the NGS Kit on the Agilent 5300 Fragment Analyzer (Agilent Technologies, Santa Clara, CA, USA) and quantified by using Qubit 4.0 Fluorometer (Invitrogen, Carlsbad, CA, USA). The sequencing libraries were multiplexed and loaded on the flow cell on the Illumina NovaSeq 6000 instrument (Illumina, Inc., San Diego, CA, USA) according to the manufacturer’s instructions. The samples were sequenced using a 2 × 150 Pair-End (PE) configuration v1.5. Image analysis and base calling were conducted by the NovaSeq Control Software v1.7 on the NovaSeq instrument (Illumina, Inc., San Diego, CA, USA). Raw sequence data (.bcl files) generated from Illumina NovaSeq were converted into fastq files and de-multiplexed using the Illumina bcl2fastq program version 2.20. One mismatch was allowed for index sequence identification.

**Computational methods:** Bulk RNA-sequencing differential expression analysis was performed in SeaPiper using R v4.1.0 [39]. RNA-sequencing reads were mapped to the human genome (GRCh38.p14) using the STAR aligner (v.2.7.10b). Prior to model fitting, low-abundance transcripts were filtered to reduce noise by excluding genes with <10 total counts across all samples and retaining only genes with ≥5 counts in at least 3 samples. Differential gene expression was performed using DESeq2 v1.32.0. *p*-values were adjusted for false discovery rate (FDR) using the Benjamini–Hochberg method. Downstream analyses were conducted within the Bioconductor framework using standard DESeq2 workflows for normalization and differential expression testing. Rlog-transformed counts were used for visualization and comparative analyses of predefined marker gene sets were performed in RStudio (v2026.01.1) using R v4.5.2. Genes not detected in the dataset were not included.

Differential expression statistics were derived from model-based log2 fold change estimates (log2FoldChange = 0.58 (~1.5×) and padj < 0.05. For Venn diagram visualization, the online tool IntersectMe was used [40]. Gene ontology (GO) terms were determined using the GOTermFinder online tool from Princeton University.

### 2.13. Gene Expression Analysis by RT–qPCR

Detailed methodology is described in the Supplementary Information, extended methods.

Samples were grouped by exposure scheme for CO (early vs. late) and processed in separate RT–qPCR runs; all samples belonging to the same scheme were analysed within the same run to enable direct comparison. Housekeeping genes were included on every plate. Relative expression was calculated using the ΔΔCt method and normalized to the corresponding control condition (organoids cultured in medium without T3 and reference THSDCs). To assess within-condition transcriptional variability, coefficients of variation (CV) were calculated from ΔCt values (normalized to UBE2D2) for three T3-responsive genes. Individual values are provided in Supplementary Table S5. Plots and statistical analyses were performed using GraphPad Prism 10.

### 2.14. LC-MS/MS

**Sample preparation:** Collected supernatants at the indicated endpoints were centrifuged at 5000 rpm for 5 min. In parallel, plastic 2 mL tubes were pre-loaded with 5 µL internal standard (IS; mixture of ^13^C_6_-T4, ^13^C_6_-T3, ^13^C_6_-rT3 and ^13^C_6_-3,3′-T2). IS was prepared as a 5-factor dilution from the previously used mixture [41]. Cleared supernatant (400 µL) was transferred into the IS-containing tubes in technical duplicate (*n* = 2). Matrix controls (NDM or COD medium, as applicable) were processed in parallel and were prepared without IS addition prior to extraction. Sample pH was adjusted by adding 5 µL of 37% HCl (Carl Roth), followed by vortex mixing. Samples were incubated in the dark at 37 °C for 30 min. Liquid–liquid extraction was performed twice using a TBME/2-propanol mixture (70% tert-butyl methyl ether (TBME; CHROMASOLV^TM^, Honeywell Honeywell Research Chemicals, 34498, Seelze, Germany) and 30% 2-propanol (CHROMASOLV^TM^, Honeywell Research Chemicals, 34965, Seelze, Germany). For the first extraction, 1000 µL of extraction solvent was added, samples were vortexed for 5 min at room temperature, and centrifuged for 5 min at 2000 rpm and 4 °C. The upper organic phase (~800 µL) was transferred to a fresh tube. A second extraction was performed by adding 900 µL fresh extraction solvent to the remaining aqueous phase, followed by vortexing and centrifugation as above. The upper organic phase was collected and combined with the first extract. After the second extraction, solvents were evaporated in a SpeedVac at 60 °C to near dryness (no visible liquid remaining). Dried extracts were resuspended in 100 µL reconstitution buffer consisting of 50% methanol (CHROMASOLV^TM^, Honeywell Research Chemicals, 34966, Seelze, Germany) and 0.1% formic acid (HiPerSolv CHROMANORM^®^; VWR Chemicals, VWRC84874.180, Radnor, PA, USA) in bidistilled water (15 MΩ·cm). Matrix controls were resuspended in 95 µL reconstitution buffer and subsequently spiked with 5 µL IS. Reconstituted samples were stored at −20 °C until LC–MS/MS analysis. All chemicals used were chromatography grade.

**LC–MS/MS:** A calibration curve was prepared in COD and NDM (6-point dilution series, with final concentrations from 0.05–50 nM (0.05, 0.25, 1.25, 5, 25, and 50 nM 3′-T1; 3,5-T2; 3,3′-T2; 3′,5′-T2; rT3; T3 and T4). The standard solutions contain equimolar 3′-T1 (LGC Standards, TRC-I721500, Teddington, Middlesex, UK); 3,5-T2 (Sigma-Aldrich, D0629, St.Louis, MO, USA); 3,3′-T2 (Sigma-Aldrich, 719536, St.Louis, MO, USA); 3′,5′-T2 (LGC Standards, TRC-D455156); rT3 (Cayman Chemical, 17598, Ann Arbor, MI, USA); T3 (Sigma-Aldrich, T6397, St.Louis, MO, USA), and L-T4 (obtained from Dr. R. Thoma; Formula GmbH Pharmaceutical and Chemical Development Company, Berlin, Germany; www.formula-pharma.de (accessed on 1 February 2026)).

Sample measurement: A HSS PFP 2.5 µm 3.0 × 100-mm column (Waters, Milford, MA, USA) maintained at 40 °C was used for separation by an 1100 HPLC system (Agilent Technologies GmbH, Waldbronn, Germany) directly coupled to a mass spectrometer QTrap 6500 (AB SCIEX Germany GmbH, Darmstadt, Germany) fitted with a Turbo Spray IonDrive.

The LC-MS/MS analysis followed the established protocols [41,42].

### 2.15. ELISA

Supernatants collected from conditions incubated with 20 nM T3 were diluted 1:5 in NDM to ensure measurements fell within the dynamic range of the assay (Supplementary Figure S10C). Wells in which NSCOs were lost during culture (empty wells) were excluded from ELISA-based T3 quantification. Supernatants were transferred to “mirror” plates, maintaining the original well coordinates, and ELISA plates were likewise arranged to preserve this coordinate system. This enabled pairing of NSCO size measurements with T3 concentrations measured in the corresponding supernatants. Total T3 concentrations were determined using a Total T3 ELISA kit (DRG, EIA-4569, DRG International, NJ, USA), following the manufacturer’s instructions. Samples were measured in technical duplicate. Mean absorbance values were calculated for standards, controls, and samples. A standard curve was fitted using a four-parameter logistic (4PL) regression model, and sample concentrations were interpolated from the fitted curve. Data analysis was performed using GraphPad Prism 10.

### 2.16. Statistical Analyses

GraphPad Prism 10 was used to generate plots. Statistical analysis was performed using RStudio (v2026.01.1) using R v4.5.2 unless stated otherwise. Data normality was assessed using the Shapiro–Wilk test and by visual inspection of residual and Q-Q plots. The definition of biological and technical replicates and the statistical tests applied for each dataset are provided in the corresponding figure legends.

### 2.17. Graphics

Schemes and graphical insets in the figures were generated using BioRender (accessed on 3 January 2026) as described in the figure legends.

### 2.18. Data Availability

RNA-sequencing data discussed in this publication is deposited in BioStudies, ArrayExpress from EMBL-EBI. Accession number: ArrayExpress accession E-MTAB-17066.

### 2.19. Declaration of AI-Assisted Technologies in the Writing Process

During the preparation of this work, the authors used ChatGPT 5.2, OpenAI (San Francisco, CA, USA), to improve readability and language. The authors reviewed and edited the content and take full responsibility for the content of the publication.

## 3. Results

### 3.1. Quality Control of hiPSC-Derived CO and NSCO Informs Batch Eligibility

Two distinct hiPSC-derived brain organoid models, namely CO and NSCO, were generated and quality-controlled to establish batch eligibility for downstream experiments. While both models are derived from hiPSCs, they differ in differentiation strategy and culture format (Figure 1A), motivating the implementation of model-specific QC criteria to ensure morphological and cellular consistency prior to experimental treatments.

COs were generated using an unguided differentiation approach developed by Lancaster et al. [25]. Successful early differentiation was defined by rosette formation observable by bright-field microscopy at day 7 and the emergence of a clearly organized neuroepithelium by day 10 following embryoid body embedding in an extracellular matrix, which supports tissue polarization (Supplementary Figure S1A,B). Bright-field microscopy was performed throughout differentiation, with day 7 and day 10 serving as predefined QC checkpoints to determine whether a batch could proceed. Additional morphology-based QC was conducted at day 20, day 40, and day 55, immediately prior to initiating experimental treatments. At these later stages, the presence of defined borders and cortical-like structures—observed at variable abundance across COs—was used as the primary criterion to retain COs for experiments. COs lacking cortical-like areas or presenting prominent cystic morphology were excluded. As an internal confirmation of bright-field-based classification, organoids were randomly sampled for histology, providing an orthogonal assessment of COs categorized as cortical unit-rich, cortical unit-containing, or morphologically abnormal/failing (Supplementary Figure S1C). Across the study, a total of 11 independent CO differentiation batches were generated from five hiPSC lines (3 different operators). Two of these batches derived from the same line (UCSFi001-A-37) were completely rejected at day 15 due to the absence of proper cortical unit formation. For the remaining lines, three batches each were generated from BIHi250-A (female) and HMGUi001-A (female), two batches from BIHi001-B (male), and one batch from BIHi005-A (male). Within accepted batches, COs exhibiting at least a small cortical-like area (approximately 10–20% of organoid structure by qualitative assessment) were retained for experiments, whereas COs with no visible cortical-like areas or cyst formation were discarded. This exclusion represented approximately 10% of the total organoids (144) generated per batch.

For the NSCOs, hiPSCs were first differentiated into NSCs using a 7-day dual SMAD inhibition protocol. NSC differentiation was performed at scale to generate NSC banks (Figure 1A and Figure S2A), thereby standardizing the starting population across independent NSCO differentiation batches. Prior to banking, day 7 NSCs were assessed by flow cytometry for expression of neural progenitor markers (SOX1, SOX2, PAX6, and NESTIN), and cryobanks were produced only for NSC differentiations with >90% positive cells for each marker (Supplementary Figure S2B). Post-thaw NSCs retained NSC marker expression by immunofluorescence (Supplementary Figure S2C), supporting bank QC and recovery. NSCOs generated for this study were derived from the same NSC banks of BIHi250-A (4 NSCO batches) and HMGUi001-A (1 NSCO batch) lines. NSCOs were subsequently generated in a 96-well plate format, supporting a medium-throughput workflow suitable for systematic exposure studies. Here, “medium-throughput” refers to manual handling of fewer than ~10–15 plates of 96-wells per experiment without liquid-handling robotics, while retaining compatibility for future automation.

### 3.2. CO and NSCO Display a Fetal-like Cortical Identity and Express Key Thyroid Hormone System Components Required for T3 Responsiveness

To establish that both organoid models provide a relevant biological context for TH system perturbation studies, we first assessed their molecular composition and expression of TH system-related genes and subsequently evaluated transcriptional responses to T3 using bulk RNA-sequencing. Because the CO and NSCO RNA-sequencing datasets were generated under different experimental designs, rlog-transformed expression values were interpreted within each model; cross-model comparisons were restricted to presence/absence patterns rather than absolute expression levels (Supplementary Table S6).

Across both models, a targeted panel of neuronal lineage markers indicated a predominantly neuroepithelial/progenitor and early neuronal state consistent with fetal-like cortical development. Progenitor markers (*SOX2*, *NES*, *PAX6*, *HES1*, *HES5*, *VIM*), outer radial glial cells (*HOPX*), intermediate progenitors (*EOMES*) and developing excitatory neuronal markers (*DCX*, *MAP2*, *NEUROD1*, *NEUROD2*) were detectable in both systems, together with forebrain/cortical identity markers (*TBR1*, *FOXG1*) (Supplementary Figure S3). Transcripts associated with neuronal layering (*SATB2* [upper-layer] and *BCL11B/CTIP* [deep-layer]) were present, consistent with ongoing glutamatergic cortical differentiation (Supplementary Figure S3). In line with the known higher-order organization of unguided brain organoids protocols, only COs displayed organized cortical units with ventricular- and subventricular zone–like cytoarchitecture, as previously described [30] (Figure 1A), whereas NSCOs did not show comparable tissue-level compartmentalization (Figure 1A). Markers of GABAergic neurons *GAD1* and *GAD2* were expressed less than excitatory neuron markers (*NEUROD1/2*, *RORB*, *SATB2*, *BCL11B*) in both models. Immature retinal (*MITF*, *RPE65*), oligodendrocyte (*OLIG2*) and astrocyte (*GFAP*) markers were less expressed in both models. Choroid plexus marker (*TTR*) and astrocyte markers showed variable expression levels in CO, in line with their heterogeneous composition (Supplementary Figure S3).

Both models expressed TH system components required for hormone transport and signalling. Transcripts encoding TH transporters (*SLC16A2/MCT8*, *SLC16A10/MCT10*, *SLC7A8/LAT2*, *SLC7A5/LAT1*) were detected both in CO and NSCO, showing a lower expression for *SLC16A10/MCT10*. *THRA* was expressed higher than *THRB* in both models, in line with the glutamatergic nature of both CO and NSCO (Supplementary Figure S3). Transporter *SLCO1C1/OATP1C1* was not detected in any of the models. Deiodinase transcripts were low (*DIO2* and *DIO3*) or absent (*DIO1*). *DIO3* transcript was upregulated in the presence of T3. In NSCO, high T3 chronic and pulse exposure induced *DIO3* expression, not seen in low T3 chronic exposure. In CO, *DIO3* expression was induced by T3 pulse exposure (Log2FC ~3; *p*-adj = 1) (Supplementary Figure S3 and Table S7). Notably, rlog values showed higher variability across technical replicates in CO compared with NSCO, likely reflecting greater organoid-to-organoid heterogeneity in CO (Supplementary Figure S3 and Table S6).

Expression of the major brain TH transporter MCT8 was further validated at the protein level in NSCO by immunofluorescence, with signal observed in both progenitor (SOX2 co-expression) and neuronal compartments (Supplementary Figure S4), consistent with prior observations in CO [30].

Together, these data show that both CO and NSCO recapitulate a fetal-like cortical and thyroid hormone-responsive molecular landscape, supporting their relevance as human in vitro models to study TH system perturbation.

### 3.3. Transcriptomic Profiling Confirms T3 Responsiveness Across Models and Supports Selection of Molecular Endpoints for THSDC Co-Exposure Studies

T3 responsiveness was evaluated by bulk RNA-sequencing following model-tailored exposure schemes (Figure 1B). COs were exposed to a 48 h T3 pulse (50 nM; “CO T3 pulse”), whereas NSCOs were subjected to either a 72 h T3 pulse (20 nM; “NSCO T3 pulse”) or continuous (“chronic”) exposure at two concentrations (1.5 nM, “chronic low T3”; and 20 nM, “chronic high T3”) (Figure 1B). These concentrations were selected to span a low-range condition approximating lower circulating total T3 and supraphysiological conditions to maximize dynamic range for transcriptomic response detection. Cerebral organoid differentiation medium (COD) and neural differentiation medium (NDM) were used as basal medium for CO and NSCO, respectively. Both COD and NDM contained 0–0.01 nM, as determined by LC-MS/MS (Figure 1D).

Differential gene expression (DGE) analyses versus matched controls (COs and NSCOs cultured in COD or NDM, respectively) showed induction of canonical T3-responsive genes across exposure conditions, with upregulation dominating the response profile (Supplementary Figure S5A and Table S7). Under pulse exposure, the highest-ranked T3-responsive genes were largely shared between CO and NSCO (Figure 1B). Notably, across all exposure conditions, a core set of consistently induced transcripts—including *HR*, *KLF9*, and *SEMA3C*—was observed, and these were combined with *DIO3* as a pragmatic molecular endpoint panel for subsequent co-exposure experiments performed in the presence or absence of reference THSDCs (Figure 1C and Supplementary Table S8).

DGE analysis (1.5× fold and *p*-adjusted value < 0.05) resulted in only nine genes (including *HR*, *KLF9* and *SEMA3C*) when NSCOs were exposed to chronic low T3 levels and in 109 and 69 for NSCOs exposed to chronic high T3 and 72 h pulse, respectively. In NSCOs, GO terms indicated that while 20 nM T3 pulse induced processes related to cell differentiation and nervous system development, a 20 nM T3 chronic exposure additionally induced immune responses related to antigen processing and presentation and interferon-mediated signalling pathway activation (Supplementary Figure S5B and Table S8). These signatures were observed despite the absence of immune cells, indicating that T3 can engage stress/inflammatory-like transcriptional programmes intrinsic to this in vitro neural system under specific exposure regimes. In CO, over a thousand genes showed 1.5× fold upregulation (including *DIO3*); however, only 29 were statistically relevant (*p*-adj value < 0.05), potentially due to a high variability between replicates (Supplementary Table S6). GO terms using upregulated genes in CO highlighted neuronal differentiation processes upon T3 pulse exposure.

Importantly, apart from the selected T3-response genes (including *DIO3*), expression of neuronal identity markers and core TH system components was largely stable across T3 exposure conditions, supporting the interpretation that T3 primarily modulated a hormone-responsive transcriptional program without overtly altering baseline neuronal lineage composition at the molecular level over the exposure windows tested.

Overall, these data demonstrate robust but exposure regimen- and model-dependent T3 responsiveness in both organoid systems and identify a focused set of molecular endpoints (*HR*, *KLF9*, *DIO3*, *SEMA3C*) suitable for THSDC co-exposure testing [13,43,44,45].

### 3.4. T3 Depletion and TH Metabolites Appearance Can Be Detected in CO and NSCO Culture Supernatant by LC–MS/MS

To calibrate a quantitative endocrine readout based on extracellular TH depletion and metabolite formation, we established an LC–MS/MS workflow to measure T3 and selected downstream metabolites in the relevant culture matrices (COD and NDM). Matrix characterization showed T3 concentrations close to the detection limit (0–0.01 nM), both in COD (*n* = 4) and NDM (*n* = 3). Using this setup, COs and NSCOs were exposed to increasing T3 concentrations for 48 h, and supernatants were analysed by LC–MS/MS (Figure 1D,E). Notably, in both models, T3 was measurably depleted from the culture supernatant, compared with matched “without organoid” controls (COD and NDM supplemented with known concentrations of T3 and incubated at 37 °C, 5%CO_2_ for 48 h). This indicates net T3 depletion from the culture supernatant over the exposure period by organoids. Importantly, T3 depletion was accompanied by the appearance of the metabolite 3,3′-T2, consistent with cellular uptake and local TH metabolism in both models (Figure 1D,E and Figure S10A). These findings align with the expression of TH system players in both models (e.g., *MCT8/SLC16A2* and *DIO3* principally) and support the concept that organoid-intrinsic processes can modulate TH availability and generate measurable metabolic products. Beyond 3,3′-T2, additional TH-related analytes were evaluated to confirm that T3 was the principal TH species in the system. 3,5-T2 was close to the lower limit of quantification (<0.05 nM) and therefore not reliably quantified, whereas 3′-T1 was detectable. T4 and reverse T3 (rT3) remained below the detection limit.

Direct quantitative comparison of T3 consumption between CO and NSCO was not performed, as organoid size, cellular proportions and model-specific metabolic capacity are not equivalent across models and were not normalized in this pilot setup.

Collectively, these measurements confirmed that T3 was the dominant TH driver in the exposure system, and that LC–MS/MS can capture both substrate depletion and metabolite formation as orthogonal, quantitative endpoints.

### 3.5. Modulation of T3 Responsive Genes Captures Reference THSDC Effects in CO and NSCO

We used a selective inhibitor of MCT8 transmembrane transporter SC and the pan-deiodinase inhibitor IA to interrogate the utility of molecular and metabolic endpoints.

**Figure 2:**
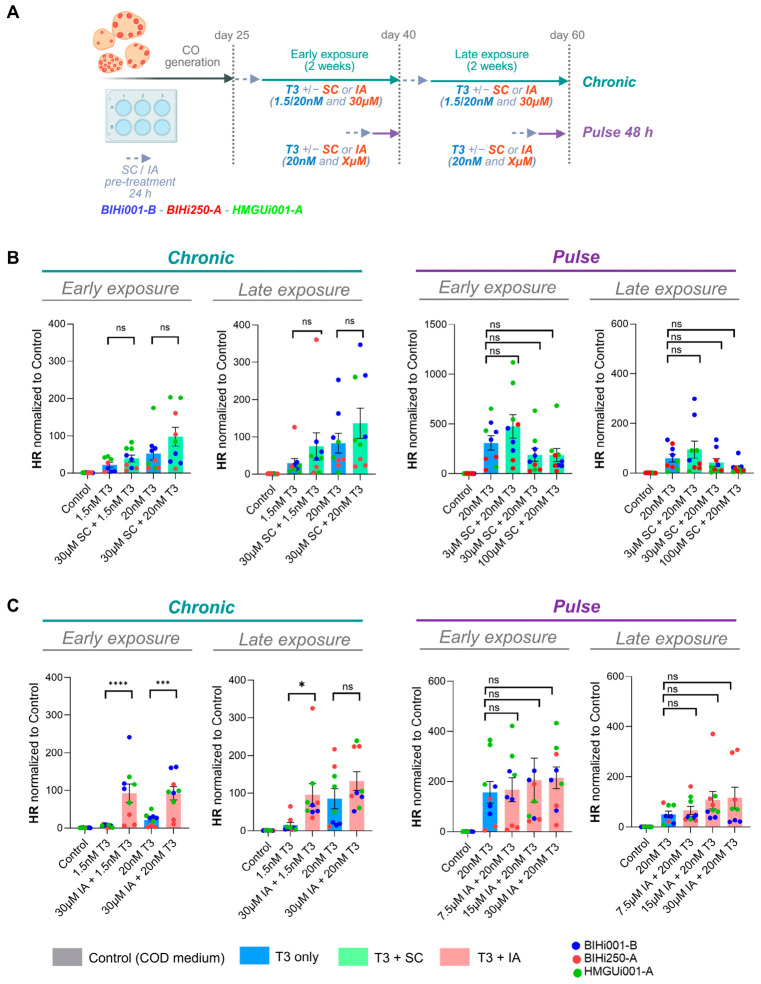
Triiodothyronine (T3)-responsive gene expression is modulated in cerebral organoids (COs) by reference thyroid hormone system-disrupting chemicals (THSDC). (**A**) Experimental design showing chronic or pulse T3 exposure combined with the reference THSDC silychristin (SC) or iopanoic acid (IA). CO were exposed during an early window (days 25–40) or late window (days 40–60), and samples were collected at day 40 (early endpoint) and day 60 (late endpoint). COs were pre-treated with SC or IA for 24 h prior to co-exposure with T3. (**B**) RT–qPCR analysis of the T3-responsive gene HR in COs co-exposed to T3 and SC, as indicated. (**C**) RT–qPCR analysis of the T3-responsive gene HR in COs co-exposed to T3 and IA, as indicated. (**B**,**C**) Data are shown as individual COs, colour-coded by hiPSC line. Bars represent mean ± SEM. Treatment groups are normalized to the respective control condition. For each condition, *n* = 3 biological replicates (hiPSC lines: BIHi001-B, BIHi250-A, HMGUi001-A) with *n* = 3 individual organoids as technical replicates per biological replicate. Statistical analysis was performed using a linear mixed effects model with treatment as the fixed effect and hiPSC line as the random effect. Comparisons were assessed using estimated marginal means with Holm correction. Significance is indicated as *p* < 0.05 (*), *p* < 0.01 (***), *p* < 0.0001 (****); ns, not significant. *Scheme in A was created using BioRender.*

CO were subjected to an experimental design with two timepoints: early (day 40, progenitor-rich state) and late (day 60, advanced cortical development) (Figure 2A). Both time points were subjected to chronic and pulse co-exposure of T3 and reference THSDC. Before co-exposure, COs were treated with SC or IA for 24 h to ensure inhibition of MCT8 and DIO3 in advance and maximize the readout effect. A panel of three T3 responsive genes (*HR*, *DIO3* and *KLF9*) was analysed in all conditions by RT–qPCR (Figure 2B, Figures S6 and S7).

Strikingly, chronic co-exposure of SC and T3 showed unexpected upregulation of T3-responsive genes independently of the CO maturation stage (early or late timepoint) (Figure 2B and Figure S6A). Chronic responses to SC effects were shown to be more defined and robust than pulse exposure. Concomitant pulse exposure to T3 and SC did not show the expected pattern (lower gene expression) or a concentration–response relationship independently of the timepoint analysed (Figure 2B and Figure S6A).

Consistent and expected upregulation of T3-responsive genes was observed upon chronic co-exposure of IA and T3, in line with T3 degradation prevention due to DIO3 inhibition, both at early and late timepoints. Transcriptomic response to pulse co-exposure was less defined without a clear pattern independently of the CO maturation stage (Figure 2C and Figure S6B).

In all genes analysed, the CO model showed high SD within conditions, as displayed by the dot-per-organoid visualization showing higher or lower CO responders. This is depicted in the plots showing individual COs coloured by hiPSC line (Figure 2B,C and Figure S6). Variability in response suggests a relation with cortical area proportion per CO, which was highly variable within CO of the same batch and between the individual hiPSC lines of origin (Supplementary Figure S7).

**Figure 3:**
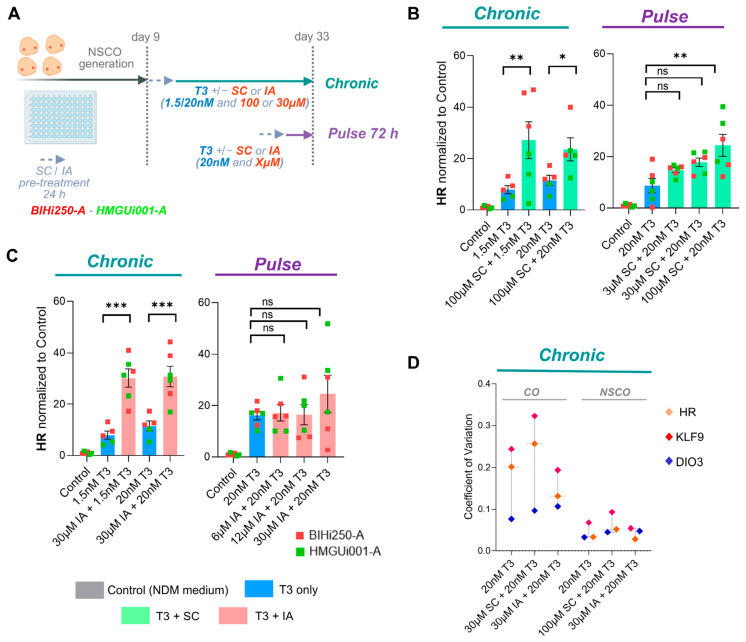
Triiodothyronine (T3)-responsive gene expression is modulated in neural stem cell-derived organoids (NSCOs) by reference thyroid hormone system-disrupting chemicals (THSDC). (**A**) Experimental design for NSCO exposure to T3 under chronic or pulse conditions in combination with the reference THSDC silychristin (SC) or iopanoic acid (IA). NSCOs were pre-treated with SC or IA for 24 h prior to co-exposure with T3, and samples were collected at day 33. (**B**) RT–qPCR analysis of the T3-responsive gene HR in NSCOs following co-exposure of T3 and SC, as indicated. (**C**) RT–qPCR analysis of the T3-responsive gene HR in NSCOs following co-exposure of T3 and IA, as indicated. (**B**,**C**) Data are shown as individual NSCOs, colour-coded by hiPSC line. Bars represent mean ± SEM. Treatment groups are normalized to the respective control condition. For each condition, *n* = 2 biological replicates (hiPSC lines: BIHi250-A and HMGUi001-A) with *n* = 3 individual NSCO pools containing 6 organoids each as technical replicates per biological replicate. Statistical analysis was performed using a linear mixed effects model with treatment as the fixed effect and hiPSC line as the random effect. Comparisons were assessed using estimated marginal means with Holm correction. Significance is indicated as *p* < 0.05 (*), *p* < 0.01 (**), *p* < 0.001 (***); ns, not significant. (**D**) Dot plot (median plus range) showing the coefficient of variation (CV) of HR, DIO3 and KLF9 expression (each dot, one gene) across replicates for matched chronic conditions in CO and NSCO (chronic 20 nM T3 alone or in combination with SC or IA, as indicated). *Scheme in A was created using BioRender.*

Experimental set up in the NSCO model was performed at one unique time point (33 days), since NSCO cytoarchitecture is not greatly modified over time, and NSCO size should remain compatible with the 96-well plate format. Chronic and pulse schemes were also used for the NSCO model (Figure 3A). Similarly to CO, NSCO chronic response to SC and IA co-exposure with T3 showed increased expression of T3-responsive genes (Figure 3B,C and Figure S8).

Concomitant exposure of T3 and SC and IA in a 72 h pulse fashion showed less consistent responses, dominated by a flat response across studied genes (Figure 3B,C and Figure S8).

Notably, NSCO exhibited greater uniformity at the phenotype level: organoid size and morphology were highly similar within condition groups (Supplementary Figure S9A,B), consistent with a more homogeneous experimental unit and supporting improved robustness of replicate-level readouts.

To better compare CO versus NSCO replicate responses, we selected *HR*, *DIO3* and *KLF9* in chronic exposure (where results were the most robust and therefore interpretable) to compare replicability across hiPSC lines and models. Within-condition transcriptional variability was assessed using the coefficient of variation (CV) across the three T3-responsive genes. While NSCO samples consistently showed lower CV compared to CO under chronic T3 exposure in the presence or not of SC and IA, this difference did not reach statistical significance, likely reflecting the limited gene panel and inter-line variability.

Together, these results show that chronic modulation of T3-responsive genes provides a more robust and interpretable readout of reference THSDC effects than pulse co-exposure, with NSCO yielding the more uniform replicate-level responses.

### 3.6. Reference THSDCs Alter Extracellular T3 Depletion and Metabolite Profiles in CO and NSCO

**Figure 4:**
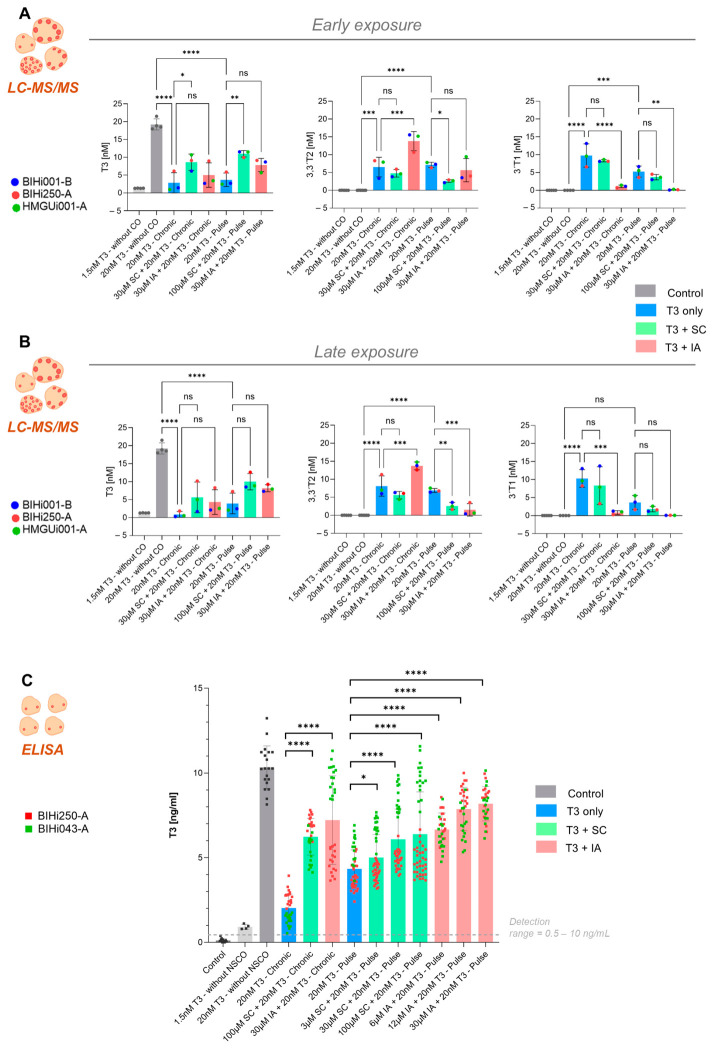
Triiodothyronine (T3) metabolism by cerebral organoids (CO) and neural stem cell-derived organoids (NSCOs) is affected by reference thyroid hormone system-disrupting chemicals (THSDC). (**A**,**B**) LC–MS/MS quantification of T3, 3,3′-diiodothyronine (3,3′-T2), and 3′-monoiodothyronine (3′T1) in CO culture supernatants collected at day 40 after early exposure (**A**) or day 60 after late exposure (**B**). Conditions include T3 exposure alone (chronic or 48 h pulse) and co-exposure with silychristin (SC) or iopanoic acid (IA) at the concentrations shown on the x-axes. Data are shown as mean ± SD with individual data points representing independent biological replicates; points are colour-coded by hiPSC line as indicated (*n* = 3 hiPSC lines: BIHi001-B, BIHi250-A, HMGUi001-A). Grey points represent measurements in wells without COs. Statistical comparisons were performed using a one-way ANOVA test followed by Tukey multiple-comparison post hoc test. Significance is indicated as *p* < 0.05 (*), *p* < 0.01 (**), *p* < 0.001 (***), *p* < 0.0001 (****); ns, not significant. (**C**) ELISA-based quantification of T3 in NSCO culture supernatants across conditions, including T3 alone and in combination with SC or IA, as indicated. Data are shown as mean ± SD with individual data points representing independent wells (one NSCO per well as technical replicates); points are colour-coded by hiPSC line as indicated (*n* = 2 biological replicates: BIHi250-A and HMGUi001-A). Black points represent measurements in wells without NSCOs. Statistical comparisons were performed using a linear mixed effects model with treatment as the fixed effect and hiPSC line as the random effect. Comparisons were assessed using estimated marginal means with Holm correction. Significance is indicated as *p* < 0.05 (*), *p* < 0.01 (**), *p* < 0.001 (***), *p* < 0.0001 (****); ns, not significant. *Schemes were created using BioRender.*

To test whether reference THSDCs modulate functional T3 metabolism, culture supernatants from T3 co-exposure experiments were quantified for T3 content and metabolite formation (Figure 4 and Figure S10A).

For CO, supernatants collected after exposure to 20 nM T3 in the presence or absence of 100 µM SC and 30 µM IA (Figure 2A) were analysed by LC–MS/MS at both early and late timepoints under chronic and pulse schemes (Figure 4A,B).

Independent of the CO maturation stage, T3 consumption in the culture supernatant decreased in the presence of SC and IA relative to the T3-only condition, under both chronic and pulse schemes (Figure 4A,B). In addition to reduced depletion, the relative distribution of metabolites differed by reference compound.

To illustrate such differences, we used the early endpoint as an example (Figure 4A). COs exposed to T3 in the chronic scheme consumed ~85% of T3 available in the medium, whereas consumption was reduced by ~30% with SC and by ~11% with IA. The metabolite fraction differed markedly across compounds: in only T3 exposure, 38% of the detected metabolite pool corresponded to 3,3′-T2 and 62% to 3′-T1; SC showed a similar split (44% 3,3′-T2, the rest as 3′-T1). In contrast, IA shifted the metabolite distribution almost entirely toward 3,3′-T2 (94%), with minimal 3′-T1 (6%) (Figure 4A). This pattern is consistent with strong interference in deiodination steps required for further conversion beyond 3,3′-T2. Under pulse exposure, culture supernatant T3 consumption dropped substantially in the presence of THSDCs (~80% vs. ~36% with SC and ~20% with IA). Importantly, under pulse conditions, the sum of measured metabolites did not fully account for the amount of T3 depleted from the medium, indicating a temporal disconnect between T3 substrate depletion and the appearance of quantified metabolites (Figure 4A). Specifically, in T3 only, exposure ~75% of the consumed T3 was recovered as quantified metabolites (43% 3,3′-T2, 32% 3′-T1), and SC co-exposure showed a similar partial recovery (~73% recovered as metabolites; 30% 3,3′-T2, 43% 3′-T1). In IA-treated organoids, metabolite recovery was lower (~50% of consumed T3 recovered as quantified metabolites), with the recovered fraction almost entirely 3,3′-T2 (48%) and very little 3′-T1 (2%) (Figure 4A). Comparable trends were observed at the late CO endpoint (Figure 4B), indicating persistence of THSDC effects across maturation stage.

For NSCO, LC–MS/MS analysis performed in pilot experiments, in a bigger format than 96-well plates, showed conversion of T3 to 3,3′-T2 and 3′-T1 and confirmed that THSDC-associated metabolite shifts mirror those observed in CO. Both SC and IA reduced net T3 consumption in culture supernatant by NSCO. For co-exposure of T3 and SC, conversion to both metabolites was observed, whereas under T3 and IA co-exposure, conversion was dominated by 3,3′-T2 with minimal 3′-T1 (Supplementary Figure S10B).

Since the T3 metabolization readout is technically incompatible with 96-well plate format, extracellular T3 depletion was quantified by ELISA as a surrogate of T3 conversion. This approach, used for the main confirmatory dataset, enabled a workflow-compatible readout for medium-throughput studies (Figure 4C). ELISA assays showed reproducible standard curves across kits (example in Supplementary Figure S10C), supporting the technical robustness of the T3 depletion measurement. Importantly, using ELISA, NSCO showed the same directional effects on T3 depletion as by LC-MS/MS (Figure 4C). Under chronic exposure, mean T3 consumption observed in the absence of THSDCs (~80%) decreased to ~40% with SC and ~30% with IA. Under pulse exposure, T3 consumption in the absence of THSDCs (~55%) decreased to ~40% with 100 µM SC and ~20% with 30 µM IA. Although absolute consumption varied between hiPSC lines, the directionality of the response to SC and IA was consistent.

Essentially, in both CO and NSCO, SC and IA reduce T3 depletion from culture supernatant, and IA reproducibly shifts the metabolite profile toward predominant 3,3′-T2 with minimal 3′-T1. Under pulse exposure, metabolite recovery lags behind T3 depletion, indicating that extracellular depletion and extracellular metabolite appearance are not strictly stoichiometric over short time windows.

### 3.7. High-Content Imaging Enables Quantification of NSCO Cell-Composition Changes

**Figure 5:**
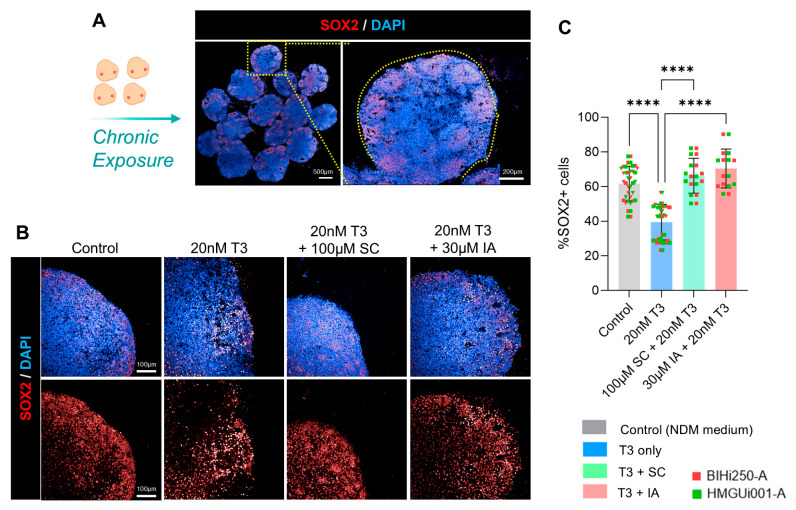
High-content imaging and analysis (HCA) enables mid-throughput quantification of histological changes following Triiodothyronine (T3) and thyroid hormone system-disrupting chemical exposure. (**A**) Representative immunofluorescence images of low magnification (overview) NSCO, stained for the neural progenitor marker SOX2 (control condition, BIHi250-A). Scale bars: 500 µm (overview) and 200 µm. (**B**) Representative immunofluorescence images showing SOX2 expression in NSCO (BIHi250-A) at the indicated different conditions upon chronic exposure to high T3 and reference THSDC. (**C**) Quantification of the percentage of SOX2-positive cells of total cell number (DAPI-positive cells) across NSCO treatment conditions. NSCOs were generated from two hiPSC lines (BIHi250-A and HMGUi001-A; *n* = 2 biological replicates); each dot represents one NSCO (technical replicate). Dots are color-coded as described, shapes depict different differentiation batches. Bars show mean ± SD. Statistical comparisons were performed using a linear mixed effects model with treatment as the fixed effect and hiPSC line as the random effect. Comparisons were assessed using estimated marginal means with Holm correction. Significance is indicated as *p* < 0.0001 (****). *Scheme was created using BioRender.*

To enable scalable, quantitative readouts of cellular composition in 3D tissue, NSCOs were embedded in an array format, allowing histological sections containing ~5–10 organoids per section, and enabling simultaneous immunofluorescence staining and automated image acquisition/analysis (Figure 5A and Figure S11A). The pipeline used a low-magnification pre-scan to detect regions of interest (ROIs) containing organoids, followed by high-magnification rescanning of each ROI for nuclei segmentation and marker classification based on nuclear morphology and fluorescence intensity. Marker-positive cells were reported as the percentage of total nuclei for each organoid and condition (Supplementary Table S4). Because NSCO sizes were highly similar within condition groups, sections could be collected at comparable tissue levels across organoids, reducing sampling-driven variability. By contrast, this was less feasible in CO due to larger size dispersion and heterogeneous internal architecture, complicating consistent region sampling.

Apoptosis was quantified using cleaved caspase-3 (cCasp3). In CO pilot analyses, cCasp3-positive nuclei ranged from ~5–25% across conditions (including different T3 concentrations and THSDC-only exposures), but high dispersion limited interpretability (Supplementary Figure S11B). This variability was consistent with regional effects within CO, where apoptosis is typically enriched in core-like areas with reduced nutrient/oxygen access, making comparisons sensitive to whether core versus peripheral regions of the COs are analysed. In NSCO, cCasp3 quantification was more robust due to their size uniformity (Supplementary Figure S11C) and therefore more comparable sampling between organoids. Overall, pulse co-exposures did not affect apoptosis across conditions, except for T3 co-exposure with 30 µM IA concentration in the pulse scheme.

Importantly, high-content imaging captured T3-dependent shifts in cellular composition that were not evident in bulk gene expression. Although SOX2 transcript levels were not modulated by T3 ± THSDCs (Supplementary Figures S3A and S12), quantification of SOX2-positive nuclei showed that 20 nM T3 decreased the proportion of SOX2+ progenitors relative to control, and this decrease was attenuated in the presence of SC or IA (Figure 5B,C). Changes in progenitor cell abundance can occur without detectable shifts in bulk *SOX2* gene expression, where individual cell gene expression cannot be analysed.

High-content imaging and analysis provide a robust, scalable way to quantify NSCO cell-state composition changes, revealing that T3 reduces SOX2+ progenitors in a manner that is mitigated by reference THSDCs.

## 4. Discussion

In vitro 3D models that recapitulate key aspects of human neurodevelopment can support the evaluation of THSDCs in a fetal-like brain context. With the aim of advancing assay development—and ultimately contributing to future validation efforts—we compared two hiPSC-derived brain organoid systems that differ substantially in complexity, scalability, and reproducibility.

### 4.1. Standardization, QC, and Assay Readiness

We established and documented generation workflows for CO and NSCO, including critical QC steps that align with emerging standards in the field [31,32,46]. A central design choice was the production and banking of intermediate NSC progenitors for NSCO generation. Banking provides a consistent starting point across experiments, improves run-to-run reproducibility, and is operationally compatible with automation, scalability and process control. In parallel, we implemented model-specific QC steps to qualify differentiation batches for downstream experimentation. Such intermediate QC is essential for organoid-based NAM workflows because it enables early identification of off-target differentiation and minimizes the risk that downstream phenotypes reflect batch failure rather than treatment effects.

### 4.2. Eligibility of CO and NSCO for TH System Studies

Transcriptomic profiling indicated that both models represent cortical-like brain regions and express key components of the TH system (including transporters, metabolic enzymes, and receptors) supporting their eligibility for TH-focused studies. As expected, CO exhibited defined cortical units and organized cytoarchitecture, consistent with prior reports [30]. In contrast, NSCO displayed neurosphere-like morphology without laminar organization.

Our findings demonstrate that a major difference between models was variability. Gene expression modulation responses were substantially more variable in CO than in NSCO. This heterogeneity is most plausibly attributed to differences in organoid composition and cytoarchitectural complexity rather than hiPSC line effects alone, although line-dependent patterns were observed in some contexts. This point is important for positioning model use: CO may be better suited for mechanistic studies that benefit from higher architectural fidelity but tolerate greater variance, whereas NSCO may be better suited for screening-style applications where robustness and scalability are prioritized.

Although COs and NSCOs show distinct characteristics that inform their suitability for assay interpretation, both models share well-recognized limitations of brain organoids. In particular, the lack of vascularization and absence of blood–brain barrier-specific properties mean that these systems cannot fully recapitulate the in vivo context of TH and THSDC uptake [47]. In turn, brain organoid immaturity (in part also driven by the lack of vascularization) constrains the study of developmental windows beyond the in vivo first trimester. In addition, brain organoids may display metabolic states that differ from endogenous neurodevelopment [48]. This limitation may affect not only the uptake and metabolism of nutrients and oxygen, but also the uptake, distribution, and metabolism of THSDCs. Therefore, while COs and NSCOs provide valuable and complementary platforms, their current biological constraints must be considered when interpreting TH system-related endpoints.

In our study, upon T3 exposure, both models exhibited a predominantly upregulated transcriptional response. Across models and exposure conditions, we identified a panel of reproducibly T3-responsive genes—*HR*, *KLF9*, *DIO3* and *SEMA3C*—which provides a practical transcriptional readout set for future studies and aligns with signatures described in prior work [13,30,43,44,45,49,50].

Among these, *DIO3* is particularly informative both as an endpoint and as a mechanistic mediator. DIO3 inactivates T3 and is highly expressed in the developing brain [51]. DIO3 expression is also increased under hyperthyroid conditions [52]. Therefore, *DIO3* upregulation likely reflects a homeostatic response to elevated T3 and must be considered when interpreting downstream outcomes, including hormone depletion and metabolite patterns in vitro.

Given the expression of *SLC16A2/MCT8* and *DIO3* as prominent TH handling components in both models, we selected SC as a selective MCT8 inhibitor and IA as a deiodinase inhibitor as reference THSDCs [9,35,36,53,54,55]. This selection was logical for establishing proof-of-principle responsiveness at the level of TH transport and metabolism.

### 4.3. Fit-for-Purpose Endpoints for THSDC Assessment

Our study shows that the combination of endpoints defined in this study (transcriptomic responses, T3 metabolism and cellular composition) is of relevance because TH system disruption can manifest at several levels and relying on a single readout risks missing relevant mechanisms. We demonstrated the need for complementary assays by showing that, although transcriptional endpoints in THSDC pulse-exposure schemes did not yield clear-cut outcomes, alterations in T3 metabolism were readily detected. Similarly, while bulk SOX2 gene expression in NSCOs was not modulated by chronic exposure to T3 or T3 plus THSDC, the number of SOX2-positive progenitor cells was affected. Together, these findings show that combining distinct endpoints is better suited to capture local tissue-specific shifts in thyroid hormone action.

A critical aspect of assay interpretability is experimental design, including concentration selection for both T3 and reference THSDCs and the exposure scheme.

Because expression of *DIO1* and *DIO2* (canonical enzymes converting T4 to T3) was very low or absent in these organoids, and *DIO3* (preferred substrate T3) was expressed, T3 was selected as the primary hormone input [53,56,57]. We therefore used a “physiological-range” concentration (1.5 nM) and a supraphysiological concentration (20 nM) to bracket conditions relevant to tissue-level exposure. While nanomolar concentrations are plausible for local tissue TH availability, brain-specific tissue concentrations remain difficult to infer from circulating levels alone, as tissue TH is shaped by transport and local deiodinase activity and may not directly correlate with serum measurements [58,59]. These uncertainties reinforce that in vitro concentration choices should be framed explicitly as operational ranges for eliciting measurable TH pathway engagement rather than direct replicas of in vivo brain exposure.

We applied two exposure schemes—chronic and pulse—to capture mechanistically distinct scenarios. Chronic exposure can influence developmental trajectories and cell composition over time, whereas a short pulse may induce transient signalling changes that may or may not propagate into stable long-term phenotypes detectable at later endpoints. Only for CO, developmental timing for exposure was particularly important because cell composition and cortical architecture evolve substantially over differentiation [30].

Our study demonstrated that in co-exposure experiments, reference THSDC effects were detectable at the gene expression level, with chronic exposures yielding fewer variable responses than pulse exposures. Intriguingly, chronic co-exposure of T3 and SC showed unexpected upregulation of T3-responsive genes, suggesting that sufficient T3 was able to enter the cells, which may be attributed to the expression of *LAT1*, *LAT2,* and to a lesser extent *MCT10*, all unaffected by SC, in line with previously described findings in neuronal cultures derived from MCT8-deficient cells [60]. Chronic co-exposure to T3 and IA showed the expected gene modulation pattern. However, this effect was not clear in the pulse scheme, where concomitant fast upregulation of *DIO3* may hamper the result in a short time. CO maturation stage in the conditions tested was not a variable to carefully consider using the endpoints described here.

A major advance of this work is the direct quantification of T3 conversion into metabolites by LC–MS/MS in both CO and NSCO. We showed that both models convert T3 into the not thyromimetically active 3,3′-T2 and 3′-T1, while conserving stoichiometry [56,57].

An important implication of these data is that both CO and NSCO support functional T3 handling that can be measured directly by the hormone concentrations in the culture supernatant rather than inferred solely from downstream transcriptional responses. In particular, combining extracellular T3 depletion with metabolite formation (e.g., 3,3′-T2) provides a mechanistically proximal endpoint that is aligned with current efforts to expand and standardize in vitro approaches for TH system disruption testing. Strikingly, in the chronic presence of IA, 3,3′-T2 was not further converted to 3′-T1. This finding is consistent with inhibition of downstream deiodination steps and provides additional evidence that LC–MS/MS profiles can distinguish disruption modes at the metabolic level.

Our work shows the potential of high-content imaging-based cell-composition analysis using an automation-compatible workflow as complementary quantitative endpoints beyond transcriptomics and hormone measurements. SOX2-expressing cell proportion changes suggested a reduction in progenitor populations under chronic T3 exposure, which was attenuated in the presence of reference THSDCs. Whether these observations correlate with changes in proliferation, differentiation, survival, or shifts in regional identity remains to be addressed.

## 5. Conclusions

Collectively, these results underscore that brain organoid standardization and reproducibility are foundational for toxicology-oriented NAM development. Intermediate QC to qualify differentiation batches and inclusion of both biological (multiple hiPSC lines) and technical replicates are essential for interpretability.

While COs provide higher architectural complexity and may be preferable for detailed mechanistic studies, NSCOs deliver more reproducible endpoints and are better aligned with automation and screening-scale workflows. In this context, reduced morphological complexity is not a limitation per se; rather, it can be an advantage when assay robustness and throughput are the primary objectives.

We have shown that combining a set of endpoints decreases the risk of missing meaningful adverse outcome pathways.

### Future Directions

Future work should critically evaluate the potential of more complex models, which partially address well-known limitations such as lack of vascularization and subsequent brain organoid maturation [61]. Improved methods will enable more robust and functional endpoints, such as neural activity assessment. Furthermore, novel technical approaches such as mosaic organoids will provide higher biological breadth under identical exposure conditions by multiplexing genetic backgrounds within the same experimental setup [60,62].

From a regulatory point of view, the outlook points to international harmonization of the NAM-specific working frame for model validation, the selection of reference THSDCs, exposure schemes and expected endpoint outcomes.

## Data Availability

RNA-sequencing data discussed in this publication will be deposited in BioStudies, ArrayExpress from EMBL-EBI upon final acceptance of the manuscript.

## Supplementary Materials

The following supporting information can be downloaded at: https://www.mdpi.com/article/10.3390/cells15110963/s1, Supplementary Information Extended materials and methods including: hiPSC lines banks and quality control (QC); Cerebral organoid (CO) generation; Immunofluorescence on Neural Stem Cells (NSC); Histology and immunofluorescence of organoids (CO and NSCO); RT-qPCR; Table S1: Antibodies; Table S2: Immunofluorescence conditions; Table S3: Primer sequences; Table S4: High content analysis (HCA) pipeline; S5: Transcriptional variability; Table S6: RNAseq_rlog; Table S7: RNAseq_DGE_all genes; Table S8: RNAseq_DGE_analysis; Figure S1: Cerebral organoid (CO) morphology provides a practical quality-control readout to identify batch differentiation heterogeneity; Figure S2: Neural stem cell (NSC) generation and quality control (QC) prior to NSCO production; Figure S3: Selected marker gene expression in neural stem cells-derived organoids (NSCO) and cerebral organoids (CO) across T3 exposure conditions; Figure S4: MCT8 expression in neural stem cell–derived organoids (NSCO); Figure S5: Differential gene expression (DGE) analyses across T3 exposure conditions in cerebral organoids (CO) and neural stem cells-derived organoids (NSCO) using bulk RNA-sequencing; Figure S6: Modulation of T3-responsive gene expression by reference thyroid hormone system–disrupting chemicals (THSDC) is highly variable across cerebral organoid (CO) replicates; Figure S7: Cerebral organoid (CO) derived from different hiPSC lines show morphological variability within the batch; Figure S8: Modulation of T3-responsive gene expression by reference THSDC across neural stem cells-derived organoid (NSCO) replicates; Figure S9: Neural stem cell–derived organoids (NSCO) display homogeneous size across replicates; Figure S10: Quantification of T3 and downstream metabolites in neural stem cell–derived organoids (NSCO) culture supernatants; Figure S11: High-content imaging workflow and analysis of cell apoptosis; Figure S12: SOX2 gene expression in neural stem cell–derived organoids (NSCO) is stable across treatments.

## Abbreviations

The following abbreviations are used in this manuscript:

2D	Two-dimensional
3,3′-T2	3,3′-diiodothyronine
3′-T1	3′-monoiodothyronine
3D	Three-dimensional
CO	Cerebral organoid
COD	Cerebral organoid differentiation medium
DGE	Differential gene expression
DIO	Deiodinase
EDC	Endocrine-disrupting chemicals
GO term	Gene ontology terminology
HCA	High-content analysis
hiPSC	Human-induced pluripotent stem cells
IA	Iopanoic Acid
IS	Internal standard
NAMs	New approach methodologies
NDM	Neural differentiation medium
NEM	Neural expansion medium
NIM	Neural induction medium
NM	Neural medium
NSC	Neural stem cells
NSCO	Neural stem cell-derived organoid
OECD	Organisation for Economic Co-operation and Development
PFAS	Per- and polyfluoroalkyl substances
QC	Quality control
rT3	3,3′,5′-triiodothyronine
T4	Tetraiodothyronine
TH	Thyroid hormones
THSDC	Thyroid hormone system-disrupting chemicals
