# Supplementary material for: Comparative Evaluation of hiPSC-Derived Brain Organoids as Platforms for Assessing Thyroid Hormone System Disrupting Chemicals
Source: Cells. 2026 May 22;15(11):963. doi: 10.3390/cells15110963 (PMC13256479; doi:10.3390/cells15110963)
Supplement: Supplementary file 1 [file cells-15-00963-s001.zip › 01_Supplementary Information_proof reading.pdf]

SUPPLEMENTARY INFORMATION

EXTENDED MATERIAL AND METHODS

hiPSC lines banks and quality control (QC)

All hiPSC lines (BIHi001-B, BIHi005-A, BIHi250-A, UCSFi001-A-37 and HMGUi001-A) were banked and subjected to comprehensive QC by BIH-CUSCO, including assessment of morphology, sterility, mycoplasma testing, karyotyping (G-banding and copy number variation analysis using SNP arrays), pluripotency (trilineage differentiation), expression of undifferentiated-state markers (NANOG, OCT3/4, SSEA4, TRA-1-60), and identity confirmation by Short Tandem Repeat (STR) profiling. Certificates of analysis are available upon request.

Cerebral organoid (CO) generation

hiPSCs at ~70% confluency were dissociated to single cells using Accutase (STEMCELL Technologies, 07920), counted, and seeded in embryoid body (EB) formation medium (STEMdiff™ Cerebral Organoid Kit, STEMCELL Technologies, 08570) supplemented with 50 µM Y-27632 (STEMCELL Technologies, 72307) at 9,000 cells per well in 96-well ultra-low attachment (ULA) round-bottom plates (Corning, 7007). All differentiation steps were performed at 37°C and 5% CO_2. To minimize cell attachment to plastic surfaces, all culture ware used from this stage onward was pre-treated with Anti-Adherence Rinsing Solution (STEMCELL Technologies, 07010) following the manufacturer's instructions. On day 5, EB formation medium was replaced with induction medium (STEMdiff™ Cerebral Organoid Kit, STEMCELL Technologies, 08570). On day 7, EB morphology was documented by brightfield microscopy (Leica DMi8). EBs were embedded in 20 µL droplets of undiluted Geltrex (ThermoFisher Scientific, A1413202) and transferred to expansion medium (STEMdiff™ Cerebral Organoid Kit, STEMCELL Technologies, 08570) under static culture conditions. On day 10, organoids were transferred to 10 cm dishes containing maturation medium (STEMdiff™ Cerebral Organoid Kit, STEMCELL Technologies, 08570) and cultured under dynamic conditions on an orbital shaker (60 rpm). At this stage, organoids were inspected for the emergence of cortical units; batches failing to show cortical unit formation were discontinued (2 out of 11 batched in this study). Medium was exchanged every 2 days. On day 21, residual Geltrex capsules, when present, were carefully removed manually using needles. For subsequent treatments, COs were either transferred to 6-well plates (all CO of early exposure group, day 23 -see CO treatment section) or maintained in 10 cm dishes in maturation medium (STEMdiff™ Cerebral Organoid Kit, STEMCELL Technologies, 08570) until use (all CO of late exposure group, day 40 -see CO treatment section). As an additional QC step, COs lacking any discernible cortical regions upon microscopic inspection at day 20 were excluded from the batch. All microscopy was performed using Leica DMi8 (Leica).

Immunofluorescence on Neural Stem Cells (NSC)

Upon thawing 50,000 viable NSCs were seeded per well of a Geltrex-coated PhenoPlate 96-well imaging plate (Revvity, 6055300) and cultured in Neural Expansion Medium (NEM) composed of 50% Advanced DMEM/F12 (ThermoFisher Scientific, 12634-010) and 50% Neurobasal Medium (ThermoFisher Scientific, 21103-049), supplemented with 2% Neural Induction Supplement (ThermoFisher Scientific, A16477-01), 1X penicil-lin/streptomycin, and 10 µM Y-27632. Cells were maintained at 37°C and 5% CO_2. After 24 h, medium was replaced with NEM without Y-27632. Cell morphology and viability were assessed 5 days post-thaw. NSCs cultured in PhenoPlate 96-well plates were fixed for 15 min at room temperature (RT) using Roti®-Histofix 4% (Carl Roth, P087.4), washed with PBS, and permeabilized with 0.3% Triton X-100 (Sigma-Aldrich, T8787) in PBS. Cells were blocked for 1 h at RT in blocking solution containing 1% bovine serum

albumin (BSA; Sigma-Aldrich, A9418), 0.1% Triton X-100, and 5% donkey serum (Merck, S30). Primary antibodies were diluted in blocking solu-tion and incubated for 2 h at RT (Table 2). For negative controls (secondary antibody only), cells were incubated with blocking solution without primary antibodies. After washing with PBS, secondary antibodies (table 1) were applied for 1 h at RT in blocking solution. Nuclei were counterstained with DAPI (1 µg/mL; ThermoFisher Scientific, 62248) for 30 min at RT in PBS. After PBS washes, plates were stored in PBS at 4°C protected from light until imaging using a wide-field fluorescence Leica DMi8 microscope.

Supplementary material and methods table S1. Antibodies

Antibody	Company, Catalogue	Dilution
**Goat anti-SOX1**	R&D Systems, AF3369	1:500
**Mouse anti-Nestin**	Millipore, MAB5326	1:100
**Goat anti-SOX2**	R&D Systems, AF2018	1:500
**Rabbit anti-MCT8**	Sigma Aldrich, ATA-HPA003353	1:200
**Guinea pig anti-DCX**	Millipore, AB2253	1:300
**Rabbit anti-cleaved Casp3**	Cell Signalling, 9661S	1:300
**Donkey anti-rabbit-AF488**	ThermoFisher Scientific, A21206	1:500
**Donkey anti-mouse-AF488**	ThermoFisher Scientific, A21202	1:500
**Donkey anti-goat-AF647**	ThermoFisher Scientific, A32849	1:500
**Donkey anti-guinea pig-AF594**	Jackson Immuno Research, 706-585-148	1:500

Histology and immunofluorescence of organoids (CO and NSCO)

Vibratome sections: fixed organoids were arranged in an array at the bottom of an embedding mold (7 × 7 × 5 mm; Electron Microscopy Sciences, 62352-07). A 7% (w/v) low-gelling agarose solution in PBS (Sigma-Aldrich, A9414), maintained at 56°C, was carefully poured over the organoids, avoiding bubble formation and disruption of organoid positioning. After solidification for 30 min at room temperature (RT), agarose blocks were sectioned at RT using a VT1200S vibratome (Leica). Sections (200 µm) were collected in PBS in 24-well plates and stored at 4°C until use.

Cryosections: fixed organoids were equilibrated sequentially in 15% and 30% (w/v) sucrose solutions in water (Sigma-Aldrich, S9378), incubating each step overnight at 4°C until organoids sank. Organoids were then arranged in an array at the bottom of embedding molds (Tissue-Tek, SA62534-15). OCT compound (Tissue-Tek, 4583) was added carefully to avoid bubbles and preserve organoid positioning. Blocks were frozen on a flat surface at −80°C and sectioned using a CryoStar NX70 cryostat (ThermoFisher Scientific). Sections (30 µm) were collected onto SuperFrost Plus slides (VWR, J1810Z) and stored at −20°C until use.

Immunofluorescence staining: immunofluorescence staining was performed on both vibratome sections and cryosections using the same reagents for each processing step; incubation times differed between section types (table 2).

Supplementary materials and methods table S2. Immunofluorescence conditions.

Step	Vibratome section	Cryosection
**1) Permeabilization**	24 hs, 4°C, rocking	1 h, RT, static
**2) Blocking**	24 hs, 4°C, rocking	1 h, RT, static

	**3) Primary antibody incubation**	48 hs, 4°C, rocking	Overnight, 4°C, static
	**4) Secondary antibody incubation**	24 hs, 4°C, rocking	2 hs, RT, static
	**5) Nuclei staining**	24 hs, 4°C, rocking	1h, RT, static
	**Washes**	3 x 30 min, 4°C, rocking	3 x 10 min, RT, static

Sections were permeabilized with 0.3% Triton X-100 (Sigma-Aldrich, T8787) in PBS and blocked in blocking buffer containing 1% bovine serum albumin (BSA; Sigma-Aldrich, A9418), 0.1% Triton X-100, and 5% donkey serum (Merck, S30). All antibody incubations were performed in blocking buffer, and all washing steps were performed in PBS. For nuclear counterstaining, DAPI was prepared at 1 µg/mL in PBS. Antibodies used are listed in table 1.

RT-qPCR

Following DNase treatment, 600 ng total RNA per sample was reverse-transcribed to cDNA using the iScript™ cDNA Synthesis Kit (Bio-Rad, 170-8891) according to the manufacturer's instructions. cDNA was diluted 1:20 in nuclease-free water, and 4 µL (corresponding to ~6 ng input RNA equivalent) was used per qPCR reaction. Quantitative PCR was performed in 384-well plates in technical duplicate using SYBR® Green PCR Master Mix (Thermo Fisher Scientific, 4309155) in a final reaction volume of 10 µL per well. Amplification was carried out on a QuantStudio 6 Flex Real-Time PCR System (Thermo Fisher Scientific). Validated primer sequences are listed in table 3.

Supplementary material and methods table S3. Primer sequences.

Target	Forward (5´→3´)	Reverse (5´→3´)
HR	TGGGTCAAGTTTGATATCCGG	AGGAAGGTTGTGGAGTTGG
KLF9	TGGCTGTGGGAAAGTCTATG	GTCTGAGCGGGAGAACTTTT
DIO3	ACTGTTGAGCTCCACTTCGG	CCTGCTGCTTCACTCCTTGA
SEMA3C	GTCCTCAGCAACCACCTACTC	GCTGGCCAGAAAACACTCAAA
SOX2	TGGCGAACCATCTCTGTGGT	CCAACGGTGTCAACCTGCAT
UBE2D2	GACTTGGGTGACTCTAGGGCA	CTGCGACGGAACTAGCTGTG
RPL13	CCTGGAGGAGAAGAGGAAAGAGA	TTGAGGACCTCTGTGTATTTGTCAA

SUPPLEMENTARY FIGURES

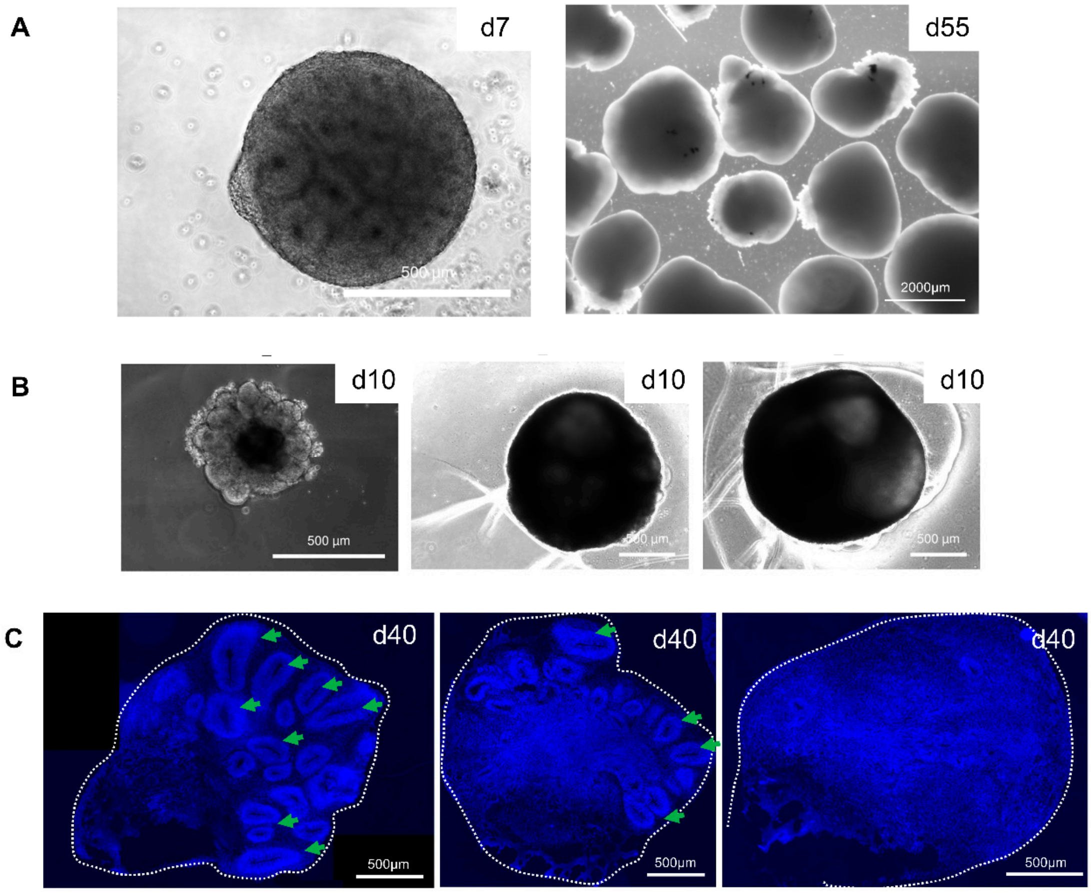

Supplementary Figure S1. Cerebral organoid (CO) morphology provides a practical quality-control readout to identify batch differentiation heterogeneity.

A) Brightfield images comparing an early-stage CO at day 7 with the corresponding day 55 CO morphology. The presence of cortical unit–like structures at early stages is shown as an indicator associated with the subsequent development of expected CO morphology. Scale bar: 500 and 2000 µm. **B)** Representative brightfield images of day 10 CO illustrating examples of expected morphology (left) and off-target morphology (middle and right), based on the presence or absence of cortical unit–like structures. Scale bars: 500 µm. **C)** Representative images of CO sections stained with DAPI from the BIHi250-A line (day 40) illustrating examples of cortical unit-rich (left), cortical unit-containing (middle) and morphologically failing (right). Cortical units are exemplified by green arrows. Scale bars 500 µm.

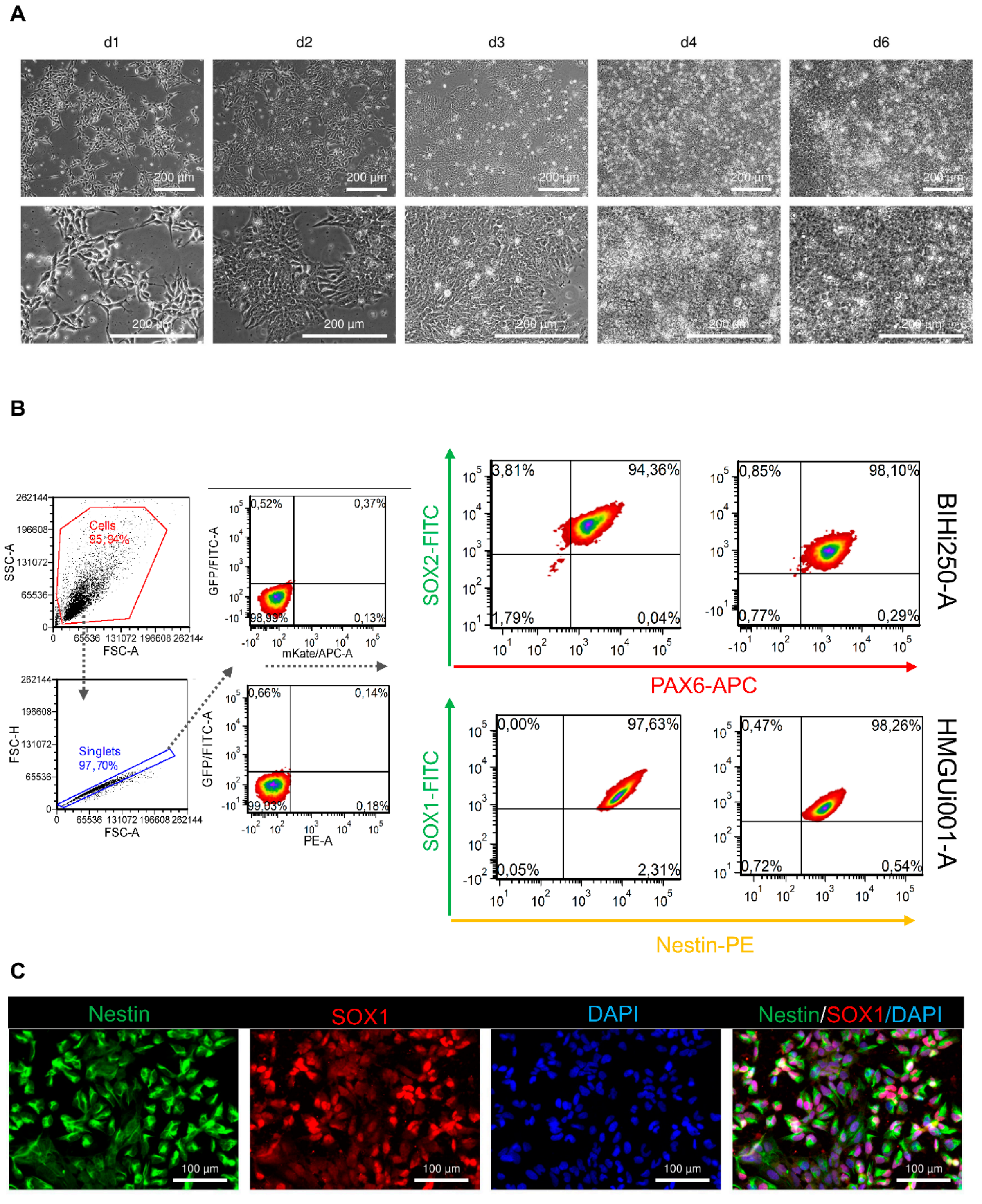

Supplementary Figure S2. Neural stem cell (NSC) generation and quality control (QC) prior to NSCO production.

A) Representative brightfield images illustrating NSC differentiation over time (days 1–6) from the BIHi250-A hiPSC line. Scale bars: 200 µm. **B)** Flow cytometry gating strategy and representative dot plots showing expression of NSC markers (SOX1, SOX2, PAX6 and Nestin) at day 7 of differentiation, prior to cryopreservation of NSC banks from two hiPSC lines used for NSCO generation in this study. **C)** Representative immunofluorescence images of thawed NSCs stained for Nestin and SOX1 with DAPI nuclear counterstain, confirming NSC identity at day 5 after recovery from cryopreservation. Scale bars: 100 µm.

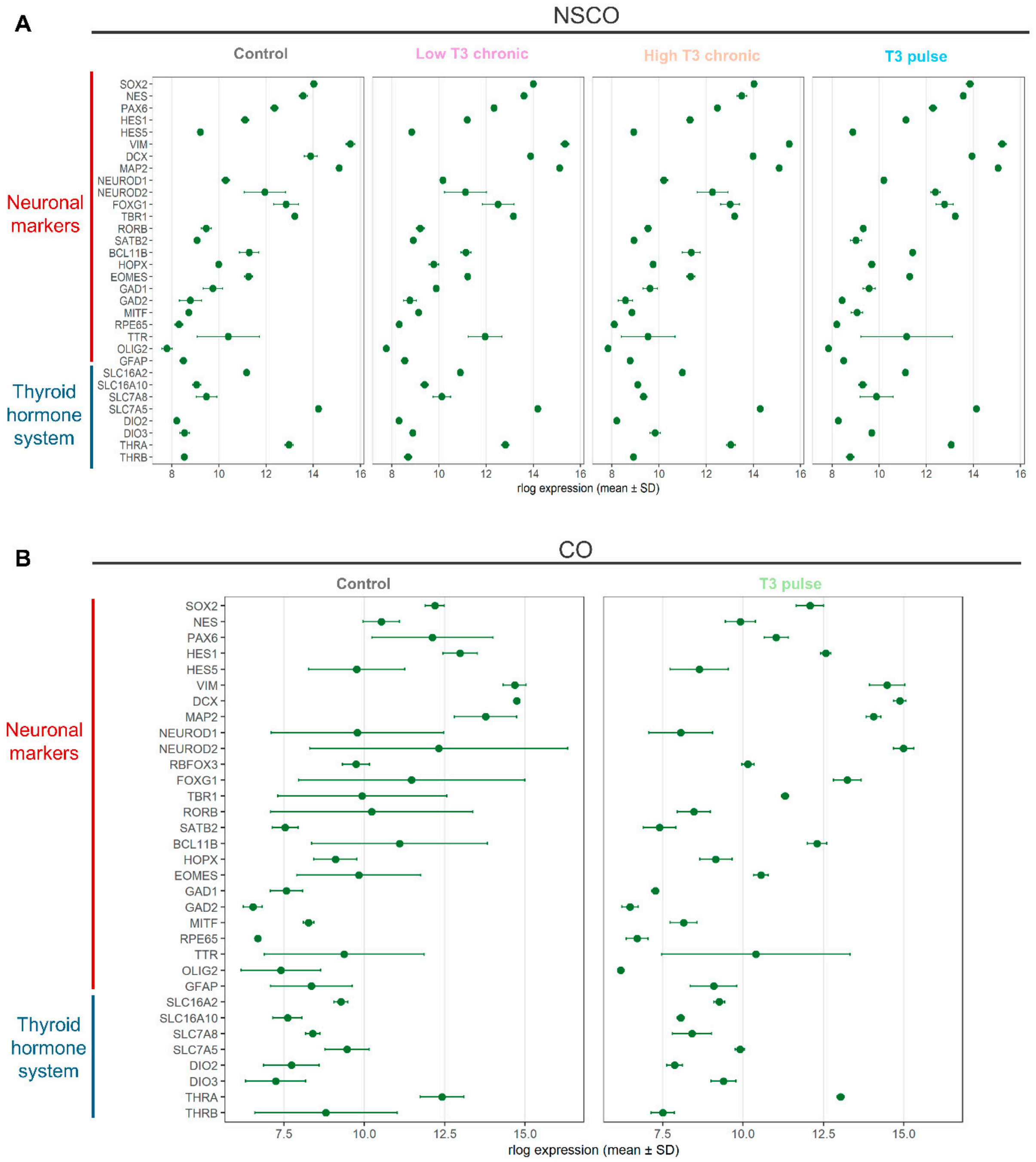

Supplementary Figure S3. Selected marker gene expression in neural stem cells-derived organoids (NSCO) and cerebral organoids (CO) across T3 exposure conditions.
Dot plots show rlog-transformed bulk RNA-seq expression values for selected neuronal identity markers and thyroid hormone transport/metabolism/signalling genes in NSCO **(A)** and CO **(B)**. For each gene and condition, points indicate the mean rlog expression across replicate samples and horizontal bars indicate ± SD. Expression status was assessed within each model independently, as NSCO and CO RNA-seq datasets were generated in separate experimental runs and are not intended for direct quantitative comparison across models. NSCO samples were derived from one hiPSC line (BIHi250-A) with n = 3 pooled samples per condition (each pool comprised 6 NSCO) **(A)** and CO samples were derived from one hiPSC line (BIHi005-A) with n = 3 pooled samples per condition (each pool comprised 3 CO) **(B)**.

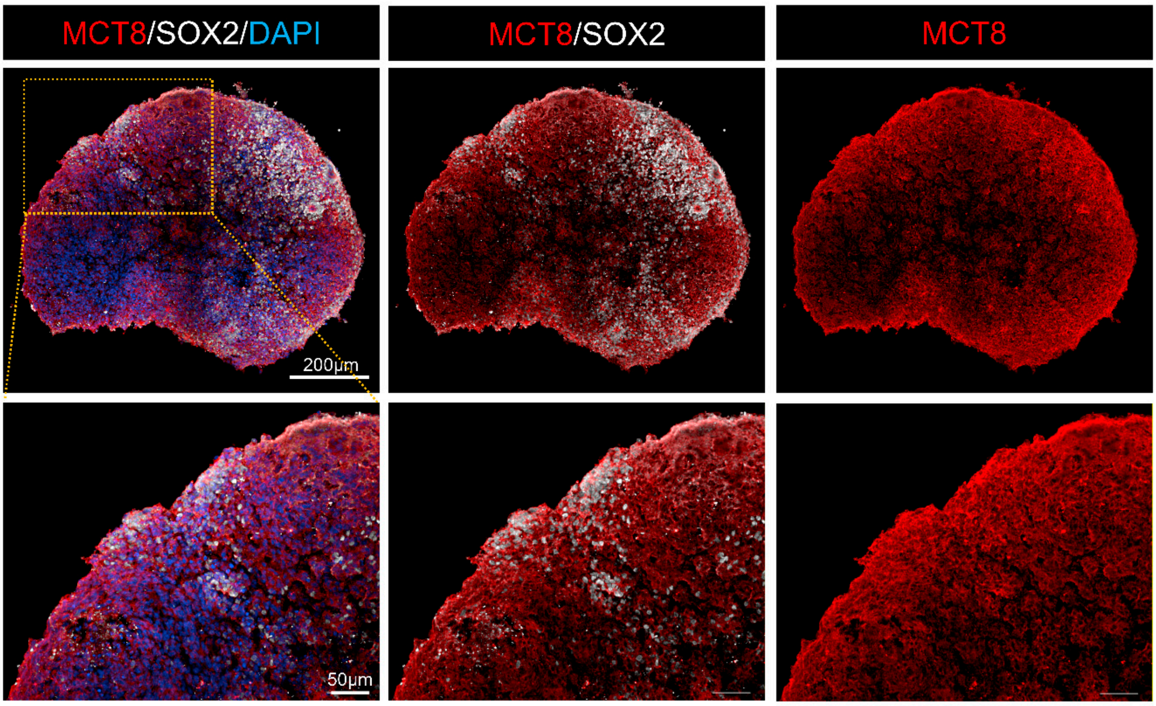

Supplementary Figure S4. MCT8 expression in neural stem cell–derived organoids (NSCO). Representative immunofluorescence images of NSCO showing expression of the thyroid hormone transporter MCT8 together with the neural progenitor marker SOX2 and DAPI nuclear counterstain. Lower panels show a higher-magnification view of the boxed region. Scale bars: 200 µm (overview) and 50 µm (higher magnification).

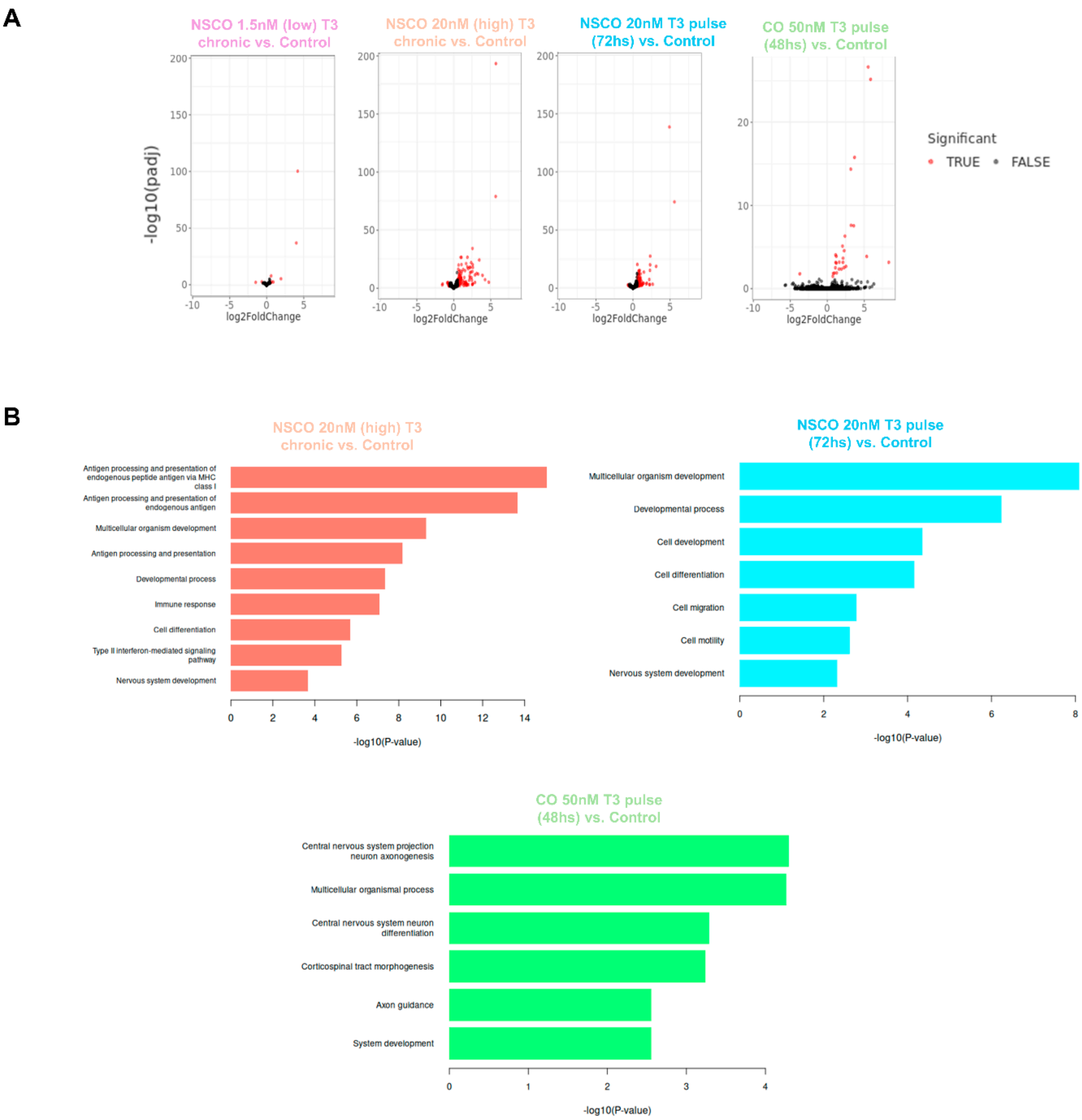

Supplementary Figure S5. Differential gene expression (DGE) analyses across T3 exposure conditions in cerebral organoids (CO) and neural stem cells-derived organoids (NSCO) using bulk RNA-sequencing.

A) Volcano plots showing DGE for each condition versus control in NSCO (1.5 nM -low- T3 chronic, 20 nM -high- T3 chronic, and 20 nM T3 pulse -72 hs-) and CO (50 nM T3 pulse -48 hs-). Points represent genes; significantly differentially expressed genes are highlighted in red. Significance thresholds were set to adjusted *p* value (padj) < 0.05 and log2 fold change ≥ 0.58 (equivalent to ≥1.5x fold change). **B)** Selected Gene Ontology terms derived from up-regulated genes (T3 treated versus control) ordered by decreasing P-value for indicated group treatments. **A-B)** NSCO samples were derived from one hiPSC line with n = 3 pooled samples per condition (each pool comprised 6 NSCO). CO samples were derived from one hiPSC line with n = 3 pooled samples per condition (each pool comprised 3 CO).

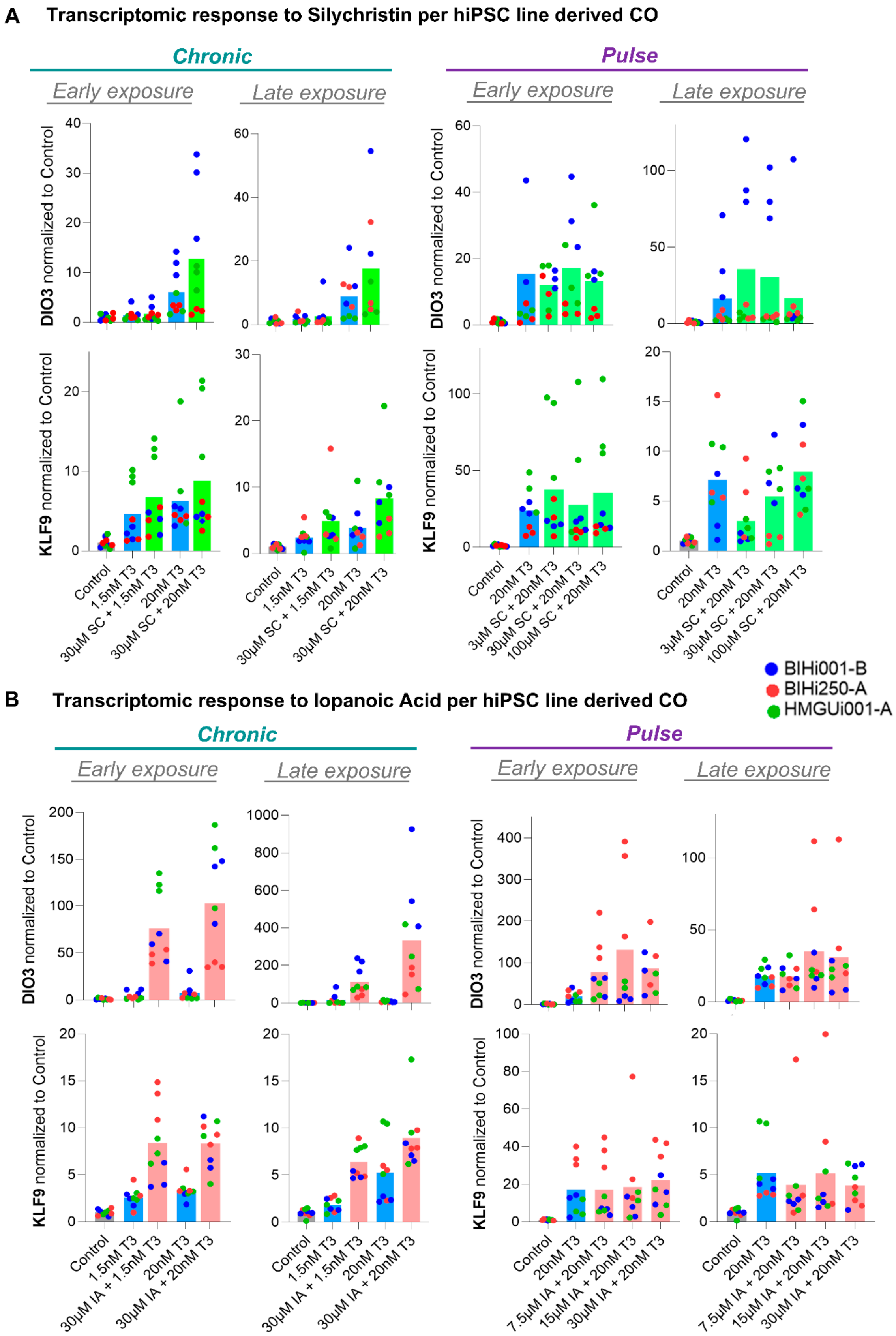

Supplementary Figure S6. Modulation of T3-responsive gene expression by reference thyroid hormone system–disrupting chemicals (THSDC) is highly variable across cerebral organoid (CO) replicates.

RT–qPCR analysis of the T3-responsive genes *DIO3* and *KLF9* in CO following chronic T3 exposure (1.5 nM or 20 nM) or a 48 hs pulse exposure in the presence or absence of silychristin (SC) **(A)** or

iopanoic acid (IA) **(B)**, as indicated. Data are normalized to the respective control condition for each condition. Bars represent the mean; points represent each an individual CO as technical replicates. CO were generated from three hiPSC lines (BIHi001-B, BIHi250-A, and HMGUi001-A), with points color-coded by line as shown.

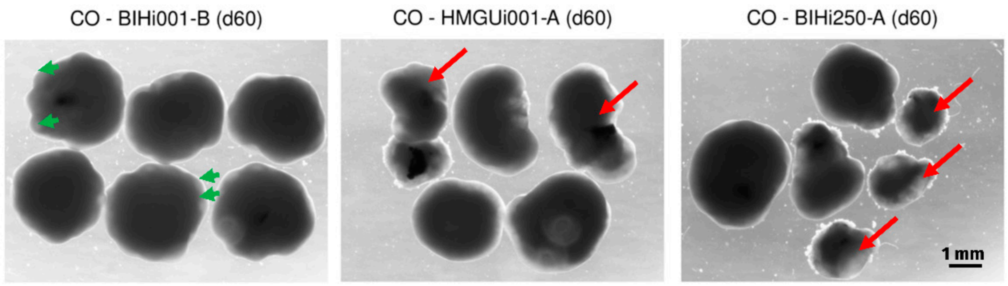

Supplementary Figure S7. Cerebral organoid (CO) derived from different hiPSC lines show morphological variability within the batch.

Representative brightfield images illustrating morphological variability among day 60 CO generated from different hiPSC lines. Green arrows indicate the presence of *cortical unit–like* structures consistent with the expected morphology, whereas red arrows highlight atypical morphologies lacking these structures. Scale bar: 1 mm.

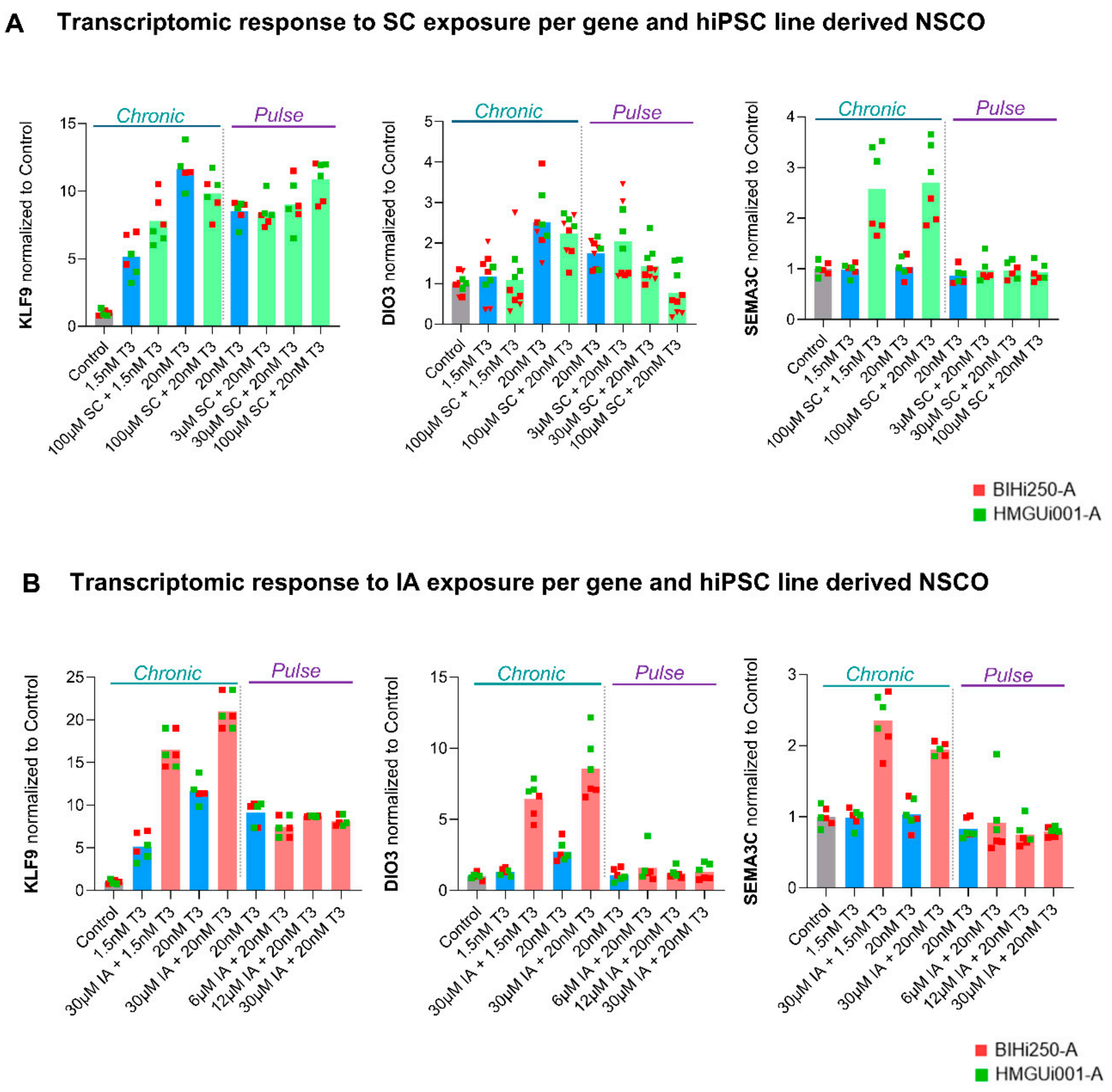

Supplementary Figure S8. Modulation of T3-responsive gene expression by reference THSDC across neural stem cells-derived organoid (NSCO) replicates.

RT–qPCR analysis of the T3-responsive genes *DIO3, KLF9* and *SEMA3C* in NSCO following chronic T3 exposure (1.5 nM or 20 nM) or a 72 hs pulse exposure in the presence or absence of silychristin (SC) **(A)** or iopanoic acid (IA) **(B)**, as indicated. Data are normalized to control condition. Bars represent the mean; points represent individual pooled samples (each point corresponds to one pool of six NSCO; technical replicates). NSCO were generated from two hiPSC lines (BIHi250-A and HMGUi001-A), with points color-coded by line as shown.

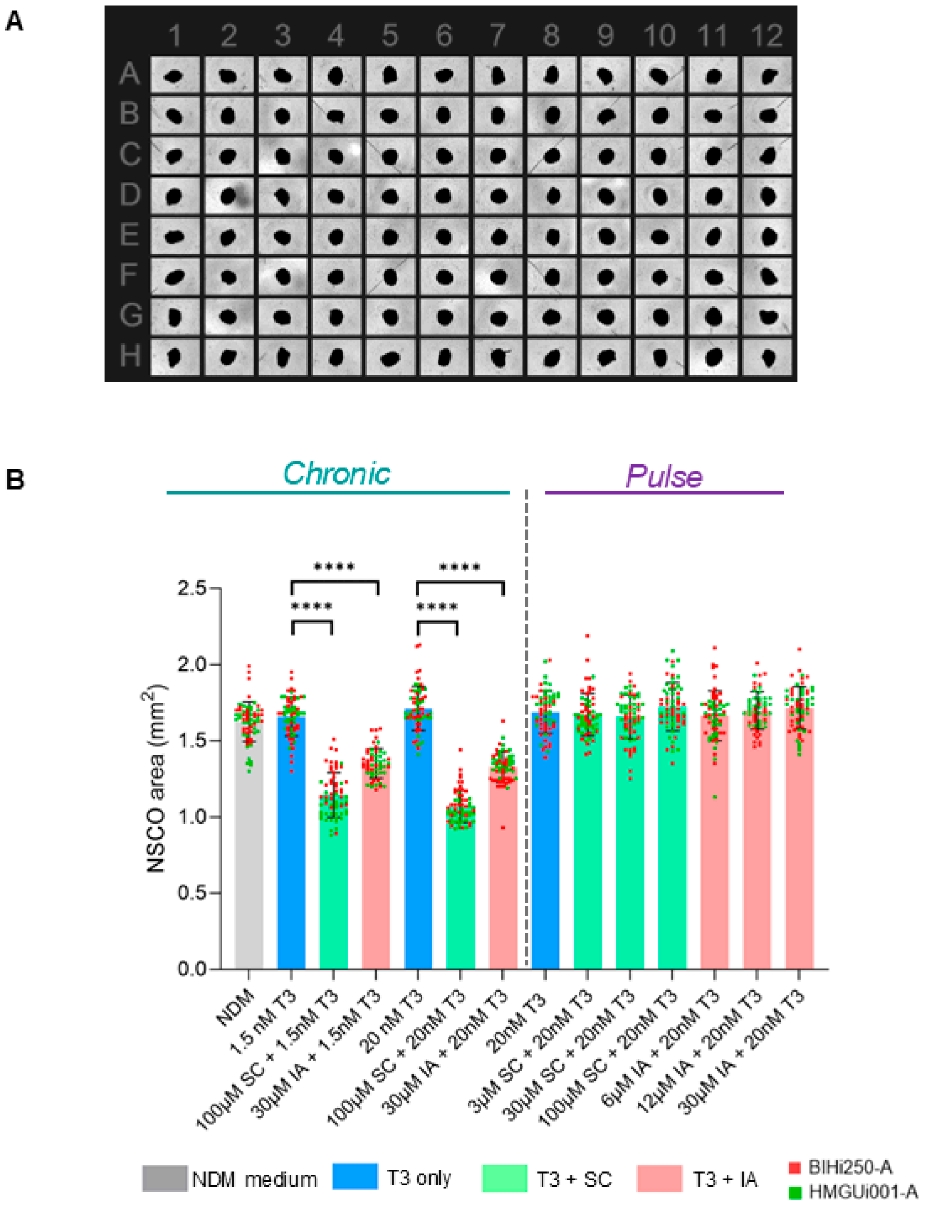

Supplementary Figure S9. Neural stem cell–derived organoids (NSCO) display homogeneous size across replicates.

A) Representative brightfield overview image of a 96-well plate containing NSCO cultured under control conditions (BIHi250-A, day 33). **B)** Distribution of NSCO area (mm^2) across treatment conditions under chronic and pulse T3 exposure, as indicated. NSCO were generated from two hiPSC lines (BIHi250-A and HMGUi001-A, biological replicates). Bars show mean ± SD; each dot represents one NSCO (technical replicates). Statistical comparisons were performed using a linear mixed effects model with treatment as fixed effect and hiPSC line as random effect. Comparisons were assessed using estimated marginal means with Holm correction. Significance is indicated as $p < 0.05$ (*), $p < 0.01$ (**), $p < 0.001$ (***), $p < 0.0001$ (****); ns, not significant.

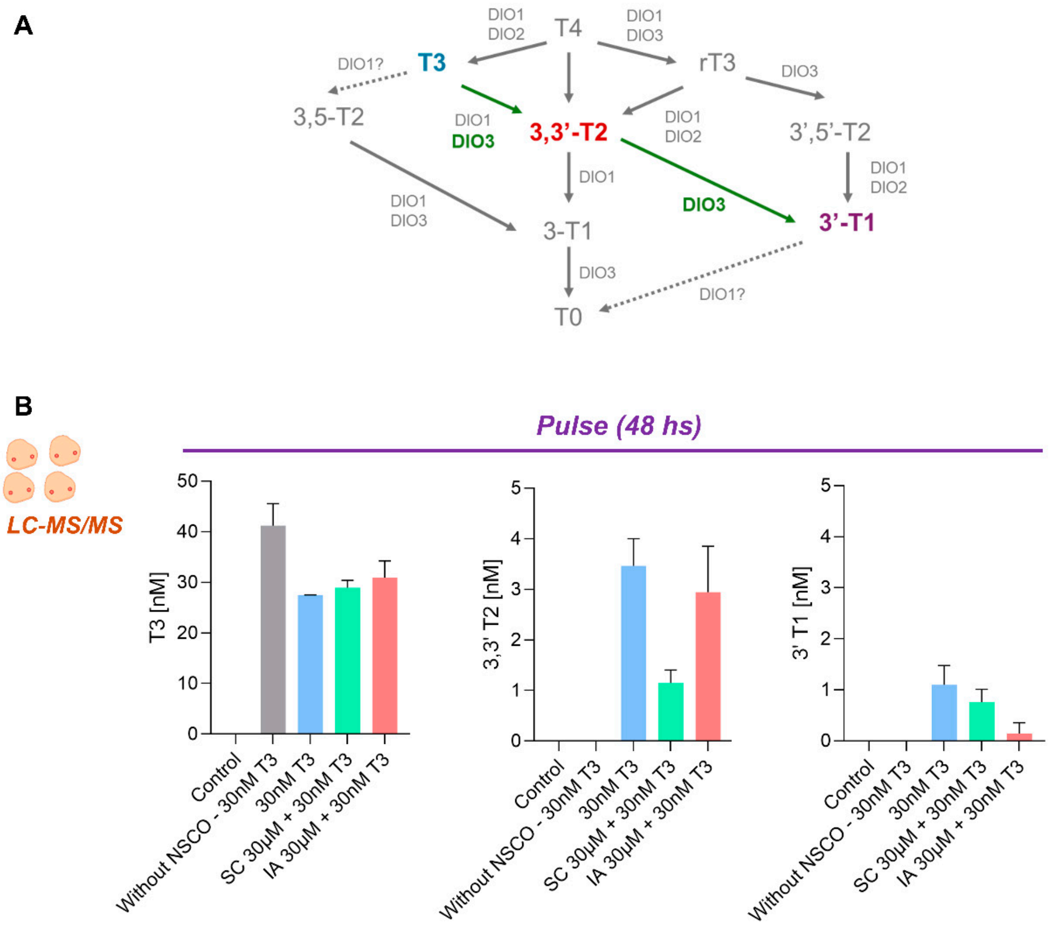

C

ELISA

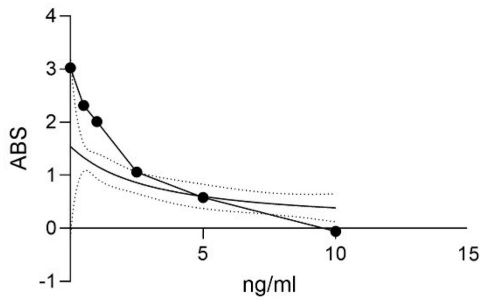

Sigmoidal, 4PL

**Goodness of Fit**	
Degrees of Freedom	2
R squared	0.99
Adjusted R squared	0.98
Sum of Squares	0.0078

Concentration correspondance

		0	ng/ml
0.77	nM	0.5	ng/ml
1.54	nM	1	ng/ml
3.85	nM	2.5	ng/ml
7.7	nM	5	ng/ml
15.4	nM	10	ng/ml

Supplementary Figure S10. Quantification of T3 and downstream metabolites in neural stem cell–derived organoids (NSCO) culture supernatants.

A) Scheme describing thyroid hormones metabolization by diodinases (DIO). **B)** LC–MS/MS quantification of T3, 3,3'-diiodothyronine (3,3'-T2), and 3-iodo-L-thyronine (3T1) in NSCO culture supernatants (BIHi250-A, day 50; 6-well format) following a 48 hs T3 pulse exposure, with or without co-exposure to silychristin (SC) or iopanoic acid (IA) at the concentrations indicated on the x-axes. Bars represent mean ± SD (n = 3 technical replicates, n=1 NSCO differentiation). **B)** Example of ELISA standard curve (standards measurements and best fit calibration curve including confidence limits -dotted lines-) and metrics used for T3 quantification in culture supernatants. Standard concentrations are indicated in ng/mL and corresponding nM concentrations.

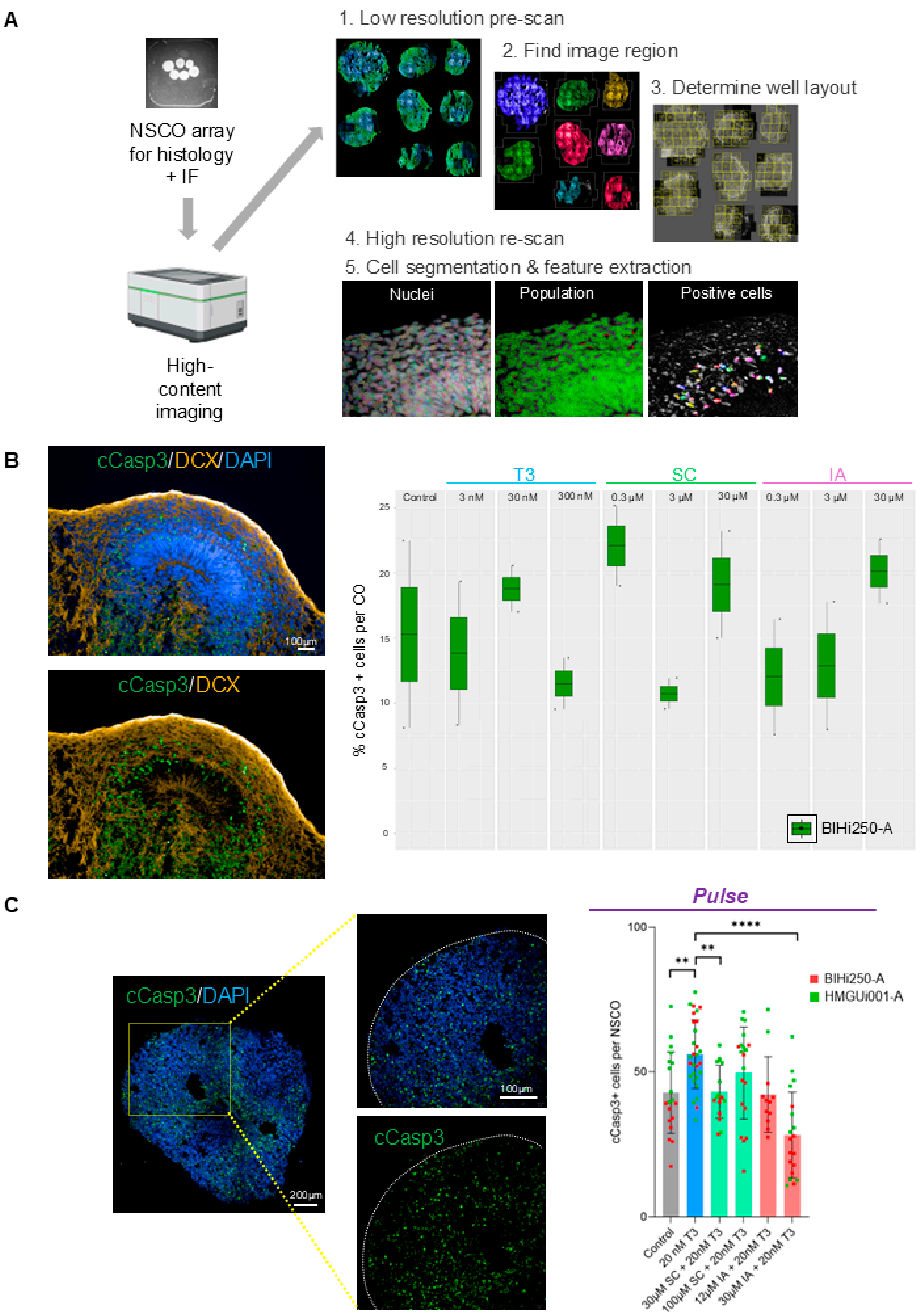

Supplementary Figure S11. High-content imaging workflow and analysis of cell apoptosis. **A)** Representative workflow for NSCO histology preparation, high-content imaging acquisition, and downstream analysis. A low-resolution pre-scan is used to localize organoids and define the imaging region and well layout, followed by high-resolution re-scanning for nuclei-based cell segmentation, population identification, and feature extraction (including nuclear morphology

parameters and mean fluorescence intensity). **B)** Representative immunofluorescence images of a cerebral organoid (CO; control condition) showing cleaved caspase-3 (cCasp3) and the early neuronal marker DCX in a cortical region (left); scale bar 100 µm. Right, boxplots showing the percentage of cCasp3-positive cells (relative to total DAPI-positive nuclei) following exposure to T3, silychristin (SC), or iopanoic acid (IA) at the indicated concentrations. Dots represent individual CO; colors indicate hiPSC line as shown. **C)** Representative immunofluorescence images of NSCO stained for cCasp3 and DAPI (left), and quantification of cCasp3-positive cells expressed as a percentage of total DAPI-positive nuclei (right). Scale bars 200 and 100 µm for overview and higher magnification, respectively. Bars show mean ± SD; dots represent individual NSCO (technical replicates) from BIHi250-A and HMGUi001-A (n = 2 biological replicates). Statistical comparisons were performed using a linear mixed effects model with treatment as fixed effect and hiPSC line as random effect. Comparisons were assessed using estimated marginal means with Holm correction. Significance is indicated as $p < 0.05$ (*), $p < 0.01$ (**), $p < 0.001$ (***), $p < 0.0001$ (****); ns, not significant.

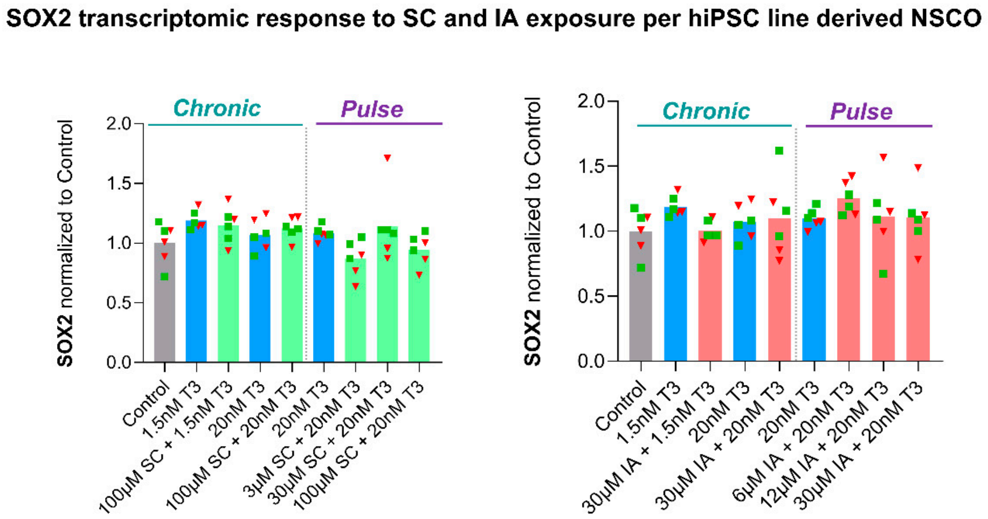

Supplementary Figure S12. SOX2 gene expression in neural stem cell–derived organoids (NSCO) is stable across treatments.

RT–qPCR analysis of neural progenitor marker SOX2 in NSCO following chronic T3 exposure (1.5 nM or 20 nM) or a 72 hs pulse exposure in the presence or absence of silychristin (SC) **(A)** or iopanoic acid (IA) **(B)**, as indicated. Data are normalized to control condition. Bars represent the mean; points represent individual pooled samples (each point corresponds to one pool of six NSCO; technical replicates). NSCO were generated from two hiPSC lines (BIHi250-A and HMGUi001-A), with points color-coded by line as shown.
