# Supplementary material for: Comparative Evaluation of hiPSC-Derived Brain Organoids as Platforms for Assessing Thyroid Hormone System Disrupting Chemicals
Source: Cells. 2026 May 22;15(11):963. doi: 10.3390/cells15110963 (PMC13256479; doi:10.3390/cells15110963)
Supplement: Supplementary file 1 [file cells-15-00963-s001.zip › 02_Supplementary Table S1_HCA Pipeline.pdf]

Analysis Sequence "cCas3 SOX2 OGD*"

Input Image	Input		
	**Channel group :** 1<br>**Sequences :** ALL<br>**Flatfield Correction :** Basic Brightfield Correction<br>**Stack Processing :** Maximum Projection		

Step	Input	Method	Output
**Filter Image**	**Channel :** Alexa 488	**Method :** Sliding Parabola<br>Curvature : 10	Output Image :<br>cCas3_SP
**Filter Image (2)**	**Channel :** Alexa 647	**Method :** Sliding Parabola<br>Curvature : 10	Output Image :<br>SOX2_SP
**Filter Image (3)**	**Channel :** HOECHST 33342	**Method :** Smoothing<br>Filter : Gaussian<br>Width : 5 px	Output Image :<br>nuclei_GS
**Filter Image (4)**	**Channel :** HOECHST 33342	**Method :** Sliding Parabola<br>Curvature : 10	Output Image :<br>nuclei_SP
**Filter Image (5)**	**Channel :** nuclei_SP	**Method :** Smoothing<br>Filter : Gaussian<br>Width : 1 px	Output Image :<br>nuclei_SP_GS
**Calculate Image**		**Method :** By Formula<br>Formula : A-B<br>Channel A : nuclei_SP<br>Channel B : nuclei_GS<br>Negative Values : Set to Zero<br>Undefined Values : Set to Zero	Output Image :<br>Calculated Image
**Find Nuclei**	**Channel :** nuclei_SP_GS<br>**ROI :** None	**Method :** C<br>Common Threshold : 0.4<br>Volume : > 120 µm³<br>Splitting Coefficient : 3	Output Population :<br>Nuclei

		Individual Threshold : 0.2<br>Contrast : > -0.24<br>Accuracy / Speed :<br>Standard / Standard	

**Calculate Morphology Properties**	**Input**	**Method**	**Output**
	**Population :** Nuclei<br>**Region :** Nucleus	**Method :** Standard<br>Volume<br>Surface Area<br>Sphericity	Property Prefix :<br>Nucleus

**Select Population**	**Input**	**Method**	**Output**
	**Population :** Nuclei	**Method :** Filter by Property<br>Nucleus Volume [μm^3] : < 2000<br>Nucleus Surface Area [μm^2] : > 150<br>Nucleus Surface Area [μm^2] : < 1500<br>Nucleus Sphericity : < 0.85<br>Nucleus Sphericity : > 0.45<br>Boolean Operations : F1 and F2 and F3 and F4 and F5	Output Population :<br>Nuclei Selected

**Select Population (4)**	**Input**	**Method**	**Output**
	**Population :** Nuclei Selected	**Method :** Common Filters<br>Remove Objects :<br>Touching Side Faces<br>Region : Nucleus	Output Population :<br>Nuclei_NSCO

**Select Region**	**Input**	**Method**	**Output**
	**Population :**<br>Nuclei_NSCO<br>**Region :** Nucleus	**Method :** Resize Region [µm/px]<br>Direction : XYZ<br>Fixed Aspect Ratio : 1<br>Outer Border XY : -2 µm<br>Outer Border Z : -2 µm<br>Inner Border XY : INF µm<br>Inner Border Z : INF µm	Output Region :<br>Nucleus Resized

**Calculate Intensity Properties**	**Input**	**Method**	**Output**
	**Channel :** Alexa 488<br>**Population :**<br>Nuclei_NSCO<br>**Region :** Nucleus Resized	**Method :** Standard<br>Mean<br>Standard Deviation<br>Coefficient of Variance	Property Prefix :<br>Intensity_cCas3

Calculate Intensity Properties (5)	Input	Method	Output
	**Channel :** Alexa 647<br>**Population :** Nuclei_NSCO<br>**Region :** Nucleus Resized	**Method :** Standard<br>Mean<br>Standard Deviation<br>Coefficient of Variance	Property Prefix : Intensity_SOX2

Select Population (3)	Input	Method	Output
	**Population :** Nuclei_NSCO	**Method :** Filter by Property<br>Intensity_cCas3 Mean : > 220<br>Intensity_cCas3 StdDev : > 40<br>Intensity_cCas3 CV [%] : > 20<br>Boolean Operations : F1 and F2 and F3	Output Population : cCas_pos

Select Population (6)	Input	Method	Output
	**Population :** Nuclei_NSCO	**Method :** Filter by Property<br>Intensity_SOX2 Mean : > 200<br>Intensity_SOX2 StdDev : > 20<br>Intensity_SOX2 CV [%] : > 15<br>Boolean Operations : F1 and F2 and F3	Output Population : SOX2_pos

Define Results	Results
	**Method :** List of Outputs<br>**Population : Nuclei_NSCO**<br>Number of Objects<br>Apply to All : ALL<br>Nucleus Volume [μm³] : ALL<br>Nucleus Surface Area [μm²] : ALL<br>Nucleus Sphericity : ALL<br>Intensity_cCas3 Mean : ALL<br>Intensity_cCas3 StdDev : ALL<br>Intensity_cCas3 CV [%] : ALL<br>Intensity_SOX2 Mean : ALL<br>Intensity_SOX2 StdDev : ALL<br>Intensity_SOX2 CV [%] : ALL<br>cCas_pos : ALL<br>SOX2_pos : ALL<br><br>**Population : Nuclei Selected**<br>Apply to All : None<br><br>**Population : Nuclei**

Apply to All : None

Population : cCas_pos
Number of Objects
Apply to All : ALL
Nucleus Volume [µm³] : ALL
Nucleus Surface Area [µm²] : ALL
Nucleus Sphericity : ALL
Intensity_cCas3 Mean : ALL
Intensity_cCas3 StdDev : ALL
Intensity_cCas3 CV [%] : ALL
Intensity_SOX2 Mean : ALL
Intensity_SOX2 StdDev : ALL
Intensity_SOX2 CV [%] : ALL

Population : SOX2_pos
Number of Objects
Apply to All : ALL
Nucleus Volume [µm³] : ALL
Nucleus Surface Area [µm²] : ALL
Nucleus Sphericity : ALL
Intensity_cCas3 Mean : ALL
Intensity_cCas3 StdDev : ALL
Intensity_cCas3 CV [%] : ALL
Intensity_SOX2 Mean : ALL
Intensity_SOX2 StdDev : ALL
Intensity_SOX2 CV [%] : ALL
cCas_pos : ALL

Method : Formula Output
Formula : a/b
Population Type : Objects
Variable a : Nuclei_NSCO - Number of Objects
Variable b : Nuclei_NSCO - Number of Objects
Output Name : fraction CTIP2 pos cells

Object Results
Population : Nuclei_NSCO : ALL
Population : Nuclei Selected : None
Population : Nuclei : None
Population : cCas_pos : ALL
Population : SOX2_pos : ALL
